# Identification of a group of bisbenzylisoquinoline (BBIQ) compounds as ferroptosis inhibitors

**DOI:** 10.1038/s41419-022-05447-8

**Published:** 2022-11-26

**Authors:** Yipu Fan, Yihan Zhang, Kunyu Shi, Shan Cheng, Duanqing Pei, Xiaodong Shu

**Affiliations:** 1 CAS Key Laboratory of Regenerative Biology, Guangdong Provincial Key Laboratory of Stem Cell and Regenerative Medicine, Guangzhou Institutes of Biomedicine and Health, Chinese Academy of Sciences, Guangzhou, 510530 China; 2 University of Chinese Academy of Sciences, Beijing, 100049 China; 3 Guangzhou Regenerative Medicine and Health Guangdong Laboratory (GRMH-GDL), Guangzhou, 510005 China; 4 School of Life Science, Westlake University, Hangzhou, 310030 China; 5 Joint School of Life Sciences, Guangzhou Institutes of Biomedicine and Health, Chinese Academy of Sciences, Guangzhou Medical University, Guangzhou, 511436 China

**Keywords:** Cell death, Drug screening

## Abstract

Ferroptosis induced by detrimental accumulation of lipid peroxides has been recently linked to a variety of pathological conditions ranging from acute tissue injuries to chronic degenerative diseases and suppression of ferroptosis by small chemical inhibitors is beneficial to the prevention and treatment of these diseases. However, in vivo applicable small chemical ferroptosis inhibitors are limited currently. In this study, we screened an alkaloid natural compound library for compounds that can inhibit RSL3-induced ferroptosis in HT1080 cells and identified a group of bisbenzylisoquinoline (BBIQ) compounds as novel ferroptosis-specific inhibitors. These BBIQ compounds are structurally different from known ferroptosis inhibitors and they do not appear to regulate iron homeostasis or lipid ROS generation pathways, while they are able to scavenge 1,1-diphenyl-2-picryl-hydrazyl (DPPH) in cell-free reactions and prevent accumulation of lipid peroxides in living cells. These BBIQ compounds demonstrate good in vivo activities as they effectively protect mice from folic acid-induced renal tubular ferroptosis and acute kidney injury. Several BBIQ compounds are approved drugs in Japan and China for traditional uses and cepharanthine is currently in clinical trials against SARS-CoV-2, our discovery of BBIQs as in vivo applicable ferroptosis inhibitors will expand their usage to prevent ferroptotic tissue damages under various pathological conditions.

## Introduction

Ferroptosis is a non-apoptotic cell death that is triggered by lethal accumulation of lipid peroxides generated from membrane polyunsaturated fatty acid (PUFA) or polyunsaturated ether phospholipids (PUFA-ePLs) by lipoxygenases or non-enzymatic Fenton reaction in an iron-dependent manner [1–3]. To defense against ferroptotic cell death, cells have evolved to use multiple antioxidant pathways to remove detrimental lipid peroxides. Among them, the GPX4/GSH pathway is the best characterized pathway that plays a central role in anti-ferroptosis defense. Disruption of this pathway by inhibition of GPX4 (by RSL3) or GSH metabolism (by erastin) is able to induce ferroptosis in a variety of cell types [1, 4]. Additional pathways such as the FSP1/CoQ10 pathway [5, 6], the GCH1/BH4 pathway [7, 8], and the DHODH pathway [9] have been recently identified to play defensive roles against ferroptosis under certain conditions. In addition, small chemical radical trapping antioxidants (RTAs) such as ferrostatin-1 and liproxstatin-1 have been developed as ferroptosis-specific inhibitors and are widely used in in vitro studies [1, 10].

Investigation of Gpx4-deficiency mice reveals multiple in vivo roles of ferroptosis in development and diseases. Gpx4 is highly expressed in the proximal tubules of kidney and inducible disruption of Gpx4 leads to ferroptotic cell death of proximal tubular epithelia and acute kidney failure in mice [10]. Ferroptosis is also observed in renal tubular cells under ischemia-reperfusion injury [11], or acute kidney injury (AKI) induced by oxalate crystal [11], folic acid [12], or cisplatin [13]. Chemotherapeutic compound such as doxorubicin or ischemia/reperfusion-induced ferroptosis is involved in cardiomyopathy as well [14]. Tissue-specific functions of Gpx4 have been investigated in additional knockout mouse lines. For example, neuron-specific knockout of Gpx4 in mice results in motor neuron degeneration and paralysis [15]. Hepatocyte-specific knockout of Gpx4 induces extensive liver degeneration [16]. Heterozygous disruption of Gpx4 in intestinal epithelial cells sensitizes mice to PUFA-rich diet-induced mucosal inflammation and enteritis [17] and ferroptosis is also involved in dextran sodium sulfate (DSS) induced ulcerative colitis [18]. In most of these abovementioned studies, inhibition of ferroptosis is generally able to ameliorate tissue damages, indicating that ferroptosis promotes the pathogenesis of these diseases.

The involvement of ferroptosis in multiple diseases promotes the development of small chemical ferroptosis inhibitors. Several RTAs have been developed as ferroptosis inhibitor thus far. While being very effective in cell culture, their activities in vivo are limited. In this study, we aimed to identify ferroptosis inhibitors with novel chemical structures and improved in vivo activities. We performed high throughput screen in an alkaloid natural compound library for compounds that are capable of inhibiting RSL3-induced ferroptosis in HT1080 cells and identified a group of BBIQ compounds as novel ferroptosis inhibitors. Several of them demonstrates promising in vivo activities and might be used as lead compounds for further anti-ferroptosis drug development.

## Results

### Identification of BBIQ compounds as ferroptosis inhibitors

**Fig. 1:**
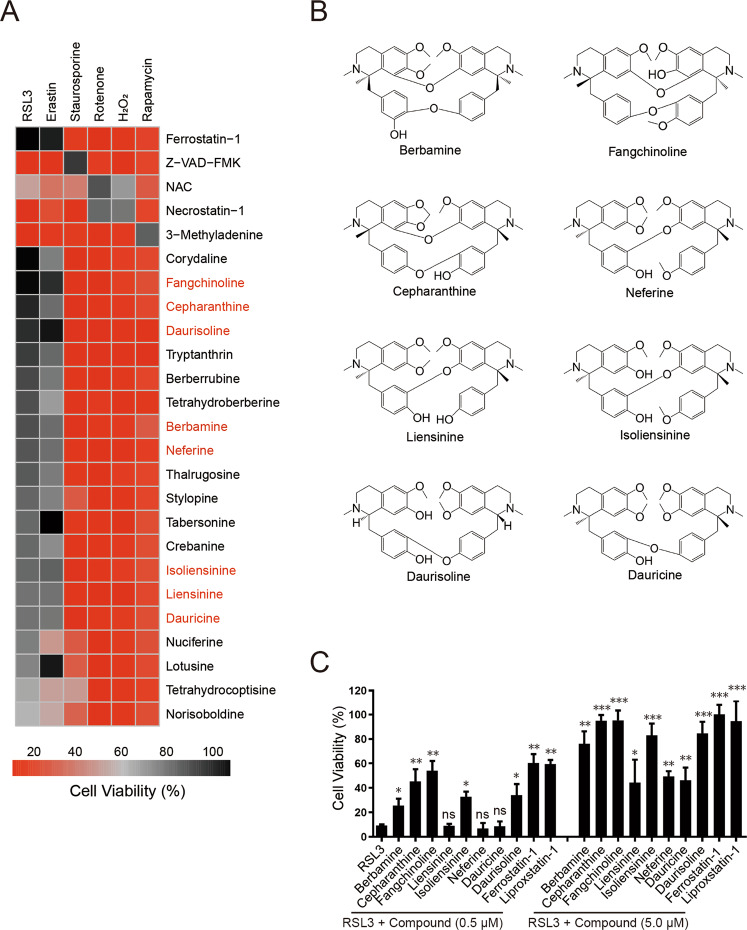
BBIQ compounds inhibit ferroptosis. **A** An overview of the top 20 hit compounds from our anti-ferroptosis screen to block cell-death induced by various stimuli in HT1080 cells. Cells were treated with RSL3 (2 µM), erastin (5 μM), staurospourine (1 μM), rotenone (3 μM), H_2_O_2_ (50 μM), or rapamycin (10 μM) in the presence or absence of a testing compound (5 µM) and cell viability analyzed at 24 h after treatment. Compounds are listed in descending order based on their rescuing activity to RSL3-induced ferroptosis. BBIQ compounds are labeled in red. **B** Structure of the eight BBIQ compounds investigated in this study. **C** Comparison of the anti-ferroptosis activities of listed compounds at 0.5 and 5 µM. Data represent mean ± s.d. from three independent experiments and *p* value (vs. RSL3 alone) is determined by ordinary one-way ANOVA with Dunnett’s multiple comparisons test. ns, no significance; *, *p* < 0.05; **, *p* < 0.01; ***, *p* < 0.001.

To identify potential small chemical ferroptosis inhibitors from natural products, we first screened an alkaloid natural compound library for compounds that can inhibit RSL3-induced ferroptosis in HT1080 cells. Cells cultured in 96-well plates were co-treated with RSL3 (2 µM) and a testing compound (5 µM) for 24 h and cell viability was then determined using the CCK-8 Kit. Assays were repeated three times (Tested compounds and their rescuing activities are listed in Supplementary Table 1) and the top 20 hits from the screen were further tested for their abilities to inhibit cell death induced by a variety of reagents (ferroptosis induced by RSL3 and erastin, necrosis induced by H_2_O_2_ and rotenone, apoptosis induced by staurosporine and autophagic cell death induced by rapamycin) (Fig. 1A). We found these compounds effectively inhibit RSL3 or erastin-induced ferroptosis but are less effective to cell death induced by other cell death stimuli tested, indicating they are ferroptosis-specific inhibitors. We analyzed the chemical structures of these compounds and found that, interestingly, eight out of the top 20 hits (berbamine, fangchinoline, cepharanthine, neferine, liensinine, isoliensinine, daurisoline, and dauricine) share similar scaffold structure (two benzylisoquionline units linked via oxygen bridge(s)) and are members of the BBIQ alkaloids (Fig. 1B). These BBIQ compounds are structurally different from known ferroptosis inhibitors such as ferrostatin-1 or liproxstatin-1 and might constitute a novel type of ferroptosis inhibitor, so we further characterized them in this study. We evaluated cellular toxicity of these compound in HT1080 cells and found they do not induce obvious toxicity at concentrations up to 10 µM while they show variable degrees of cytotoxicity at 15 µM or higher concentrations), which is generally in between ferrostatin-1 and liproxstatin-1, two widely used ferroptosis inhibitors (Supplementary Fig. 1A). We then analyzed dose response of these BBIQs to block ferroptosis and found that cepharanthine or fanchinoline at 0.5 µM is sufficient to inhibit RSL3-induced ferroptosis, while other compounds need higher dose (5–10 µM) to effectively inhibit ferroptosis (Fig. 1C and Supplementary Fig. 1B). We then tested their anti-ferroptosis activities in additional cell lines and found they also effectively inhibit RSL3-induced ferroptosis in cell lines such as lung carcinoma cell line H1975, glioma cell line U251, breast adenocarcinoma cell line MDA-MB-231 and hepatocellular carcinoma cell line HepG2 (Supplementary Fig. 2). Together, these results suggest BBIQs as a novel class of ferroptosis inhibitors.

### BBIQs block RSL3-induced accumulation of lipid ROS

**Fig. 2:**
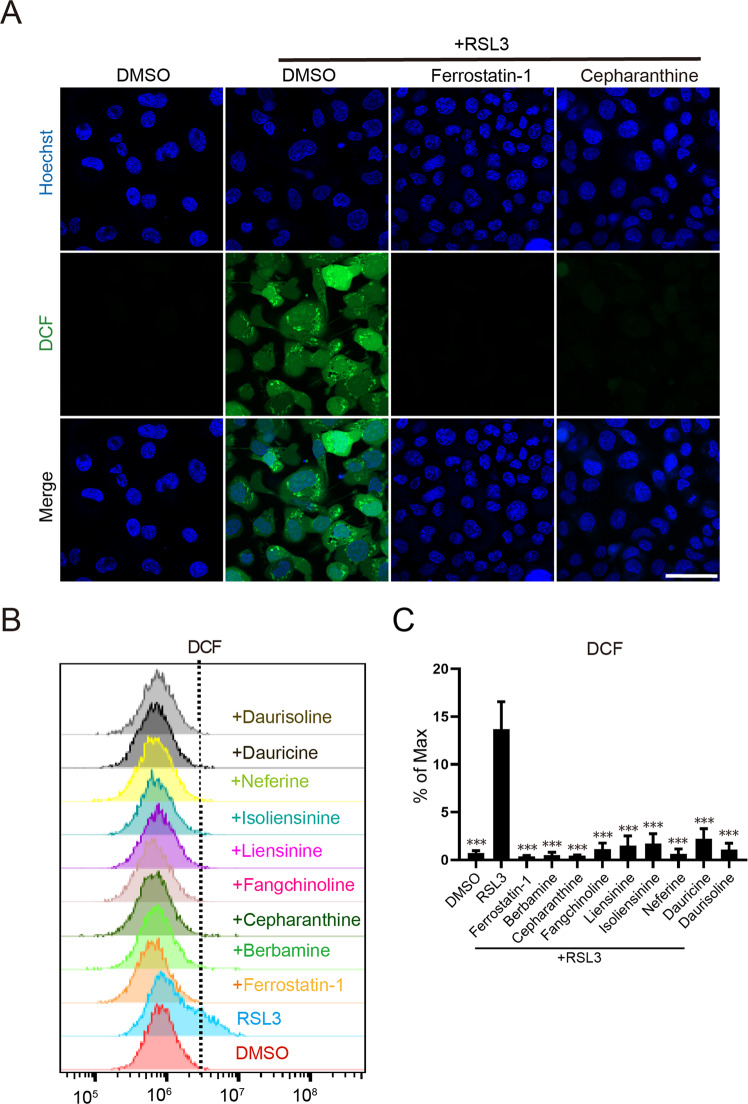
Analysis of RSL3-induced cytosolic ROS by DCFH-DA (DCF) staining. **A** Representative images of cytosolic ROS immunofluorescence staining in HT1080 cells. Cells cultured on coverslips were treated with RSL3 (2 μM) plus a testing compound (5 μM) or ferrostatin-1 (5 μM) for 4 h, labeled with the DCFH-DA probe (10 μM for 30 min) then cytosolic ROS levels were analyzed by confocal imaging. Scale bar: 50 μm. **B** Cytosolic ROS assessed by flow cytometry. Cells were treated as in (**A**) then analyzed using the BD Accuri C6 Plus Flow Cytometer. **C** Statistical results for (**B**). Data represent mean ± s.d. from three biological repeats and *p* value (vs. RSL3 alone) is determined by ordinary one-way ANOVA with Dunnett’s multiple comparisons test. ***, *p* < 0.001.

**Fig. 3:**
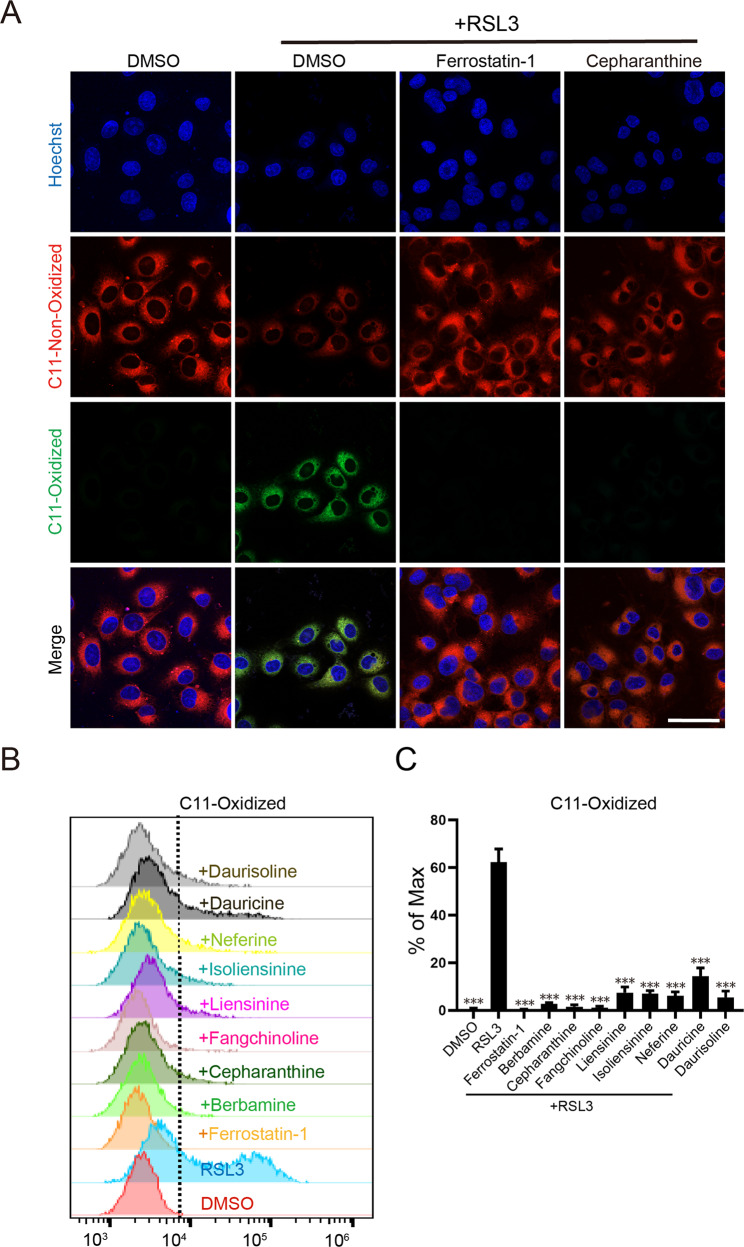
Analysis of RSL3-induced lipid ROS by C11-BODIPY 581/591 staining. **A** Representative images of immunofluorescence staining of lipid ROS. Cells were treated as described in Fig. 2A then stained with the C11 BODIPY 581/591 probe (2 μM for 20 min) and analyzed by confocal imaging. Scale bar: 50 μm. **B** Flow cytometry analysis of RSL3-induced oxidation of C11-BODIPY. **C** Statistical results for (**B**). Data represent mean ± s.d. from three independent repeats. *p* value (vs. RSL3 alone) is determined by ordinary one-way ANOVA with Dunnett’s multiple comparisons test. ***, *p* < 0.001.

**Fig. 4:**
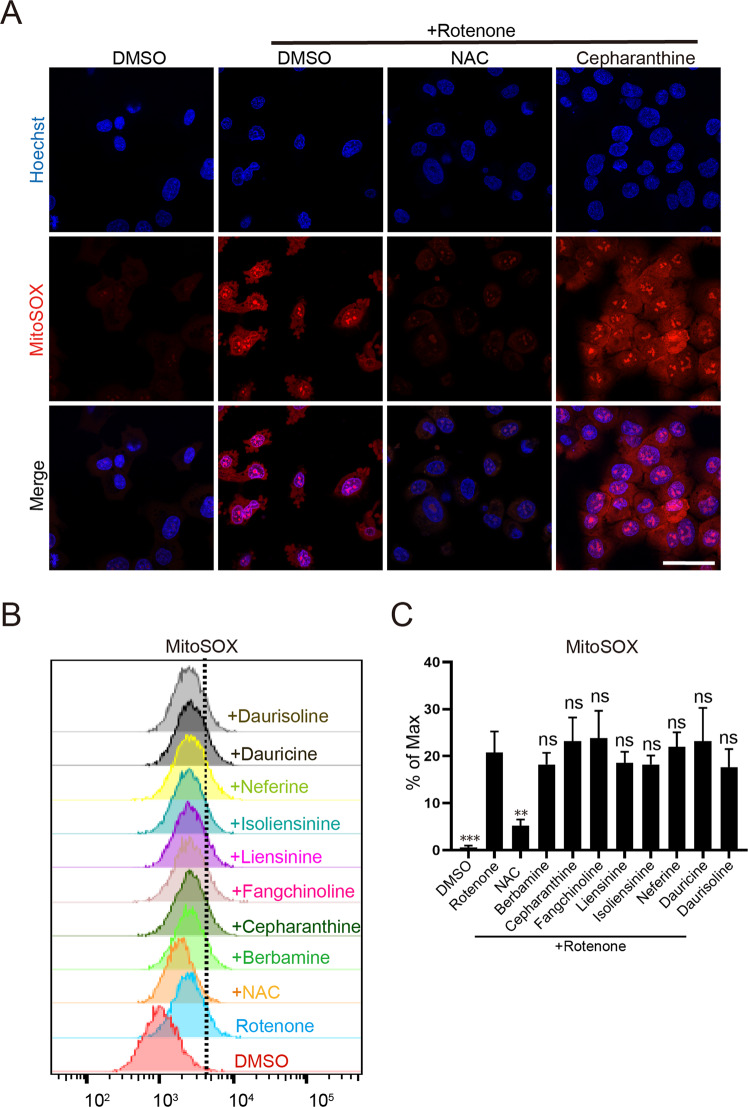
Analysis of rotenone-induced mitochondrial ROS by MitoSOX staining. **A** Representative immunofluorescence staining of mitochondrial ROS. Cells were treated with rotenone (2 μM) in the presence of a testing BBIQ compound (5 μM) or NAC (5 mM) for 4 h, stained with the MitoSOX Red Mitochondrial Superoxide Indicator (5 μM for 10 min) then analyzed by confocal imaging. Scale bar: 50 μm. **B** Flow cytometry analysis of rotenone-induced mitochondrial ROS. **C** Statistical results for (**B**). Data represent mean ± s.d. from three independent repeats and *p* value (vs. rotenone alone) is determined by ordinary one-way ANOVA with Dunnett’s multiple comparisons test. ns, no significance; **, *p* < 0.01; ***, *p* < 0.001.

Lipid peroxidation is a hallmark of ferroptosis and known ferroptosis inhibitors generally function to reduce the accumulation of detrimental lipid peroxides by either blocking lipid peroxidation (such as iron chelators) or functioning as radical-trapping antioxidants (such as ferrostatin-1 and liproxstatin-1). In previous studies, many BBIQ family compounds have been reported to modulate cellular ROS levels (either decrease or increase ROS level depending on samples analyzed). We speculated that BBIQs might regulate the level of various ROS species during RSL3-induced ferroptosis. To test this hypothesis, we first analyzed cytosolic ROS level upon RSL3 treatment. HT1080 cells were treated with RSL3 in the presence or absence of BBIQ compound for 4 h then cellular ROS levels were determined by DCFH-DA immunofluorescence staining and FACS analysis. We found that RSL3 treatment elevates cytosolic ROS level while co-treatment with ferrostatin-1 (positive control), cepharanthine, or other BBIQs all block the induction of cytosolic ROS (Fig. 2A and Supplementary Fig. 3A). FACS analysis confirmed the suppression of RSL3-induced cytosolic ROS by BBIQs (Fig. 2B, C). We next analyzed lipid ROS levels in those treated cells by C11-BODIPY staining and found that these BBIQs effectively block RSL3-induced accumulation of lipid ROS (Fig. 3 and Supplementary Fig. 3B). On the other hand, we found that BBIQs are not able to block rotenone-induced mitochondrial superoxide (detected by MitoSOX staining) (Fig. 4 and Supplementary Fig. 3C). Together, these results indicate that BBIQs inhibit ferroptosis through their downregulation of lipid ROS.

### BBIQs have intrinsic ROS scavenging activities

**Fig. 5:**
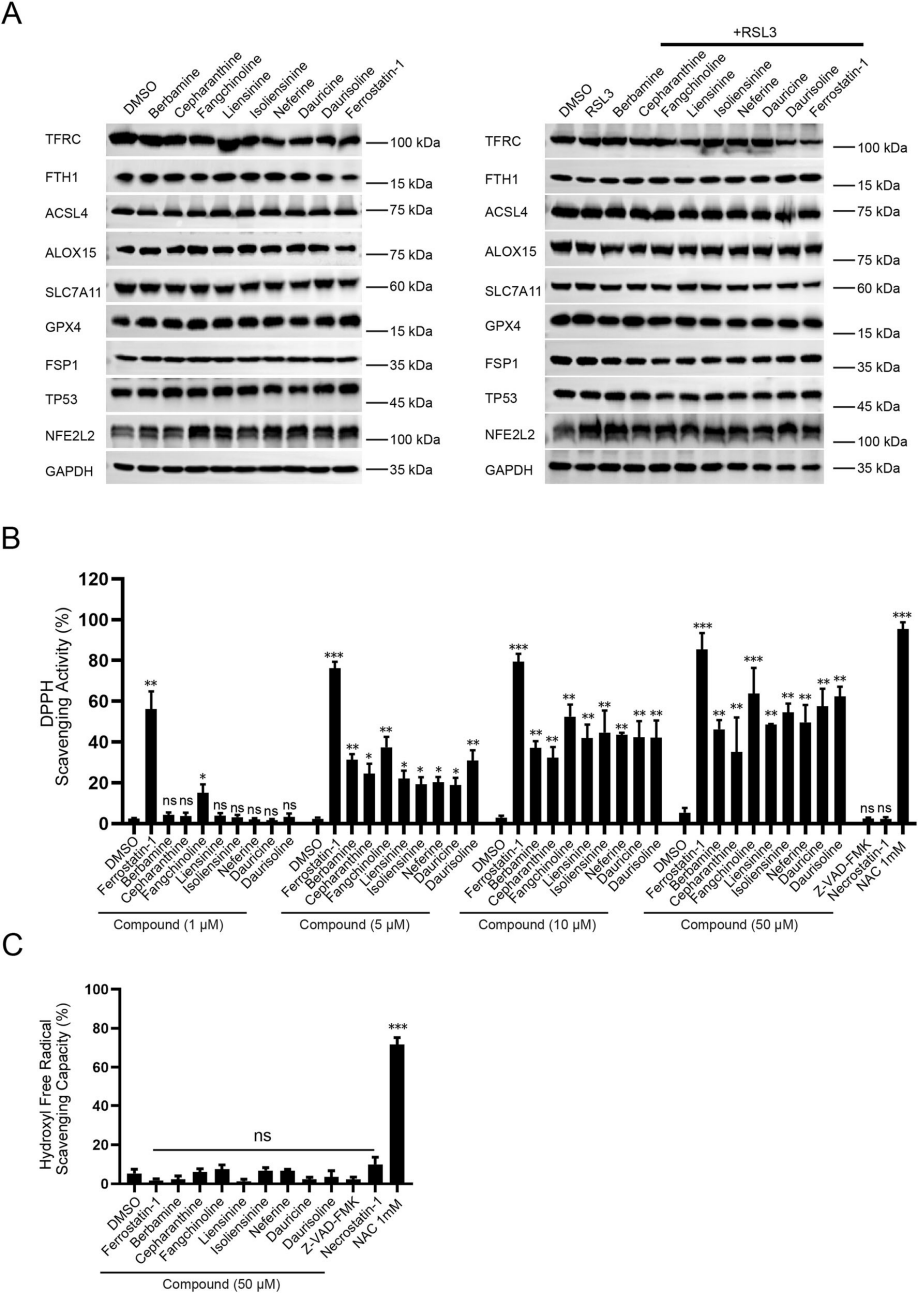
ROS scavenging activities for BBIQs in cell-free assays. **A** Representative western blots for key ferroptosis regulators. BBIQ compounds do not affect the homeostasis of all proteins examined in either unstimulated or RSL3-stimulated HT1080 cells. Assays were repeated twice with similar results. **B** Cell-free DPPH free radical scavenging assay. BBIQs were tested at 1, 5, 10, and 50 μM. Z-VAD-FMK and necrostatin-1 were tested at 50 μM and NAC at 5 mM. Data represent mean ± s.d. from three independent repeats and *p* value (vs. DMSO) is determined by ordinary one-way ANOVA with Dunnett’s multiple comparisons test. ns, no significance; **, *p* < 0.01; ***, *p* < 0.001. **C** Cell-free hydroxyl radical scavenging capacity assay. The concentration for NAC is 5 mM. Data represent mean ± s.d. from three independent repeats and *p* value (vs. DMSO) is determined by ordinary one-way ANOVA with Dunnett’s multiple comparisons test. ns, no significance; ***, *p* < 0.001.

Previous studies have identified multiple pathways involved in the homeostasis of lipid ROS and regulation of ferroptosis. We analyzed whether or not BBIQs modulate ferroptosis through these known pathways. We first determined protein levels of key ferroptosis-related factors by western blot and found that, either in the unstimulated or RSL3-stimulated cells, protein levels of key factors involved in iron metabolism (TFRC, FTH1), lipid synthesis and peroxidation (ACSL4, ALOX15), antioxidant defense (SLC7A11, GPX4, FSP1, NFE2L2) and additional ferroptosis regulator such as TP53 are not affected by BBIQ treatment (Fig. 5A). These results indicate that BBIQs do not block ferroptosis through their regulation of these pathways. We then investigated whether BBIQs have intrinsic antioxidant activities and can act as RTAs to remove ROS in cell-free assays. We found that these BBIQs (at 5, 10, or 50 µM) all have radical scavenging activities to the stable free radical DPPH in cell-free reactions (Fig. 5B). On the other hand, in a hydroxyl free radical scavenging capacity assay, we found that BBIQs (50 µM) are not effective in reducing hydroxyl free radical generated from H_2_O_2_ through Fenton reaction (Fig. 5C). Together with the observation that BBIQs do not protect cells from H_2_O_2_-induced cell death (Fig. 1A), these data indicate that BBIQs function preferentially as RTAs for lipid ROS which underlies their anti-ferroptotic activities.

### BBIQs protect folic acid-induced acute kidney injury (FA-AKI) in mice

**Fig. 6:**
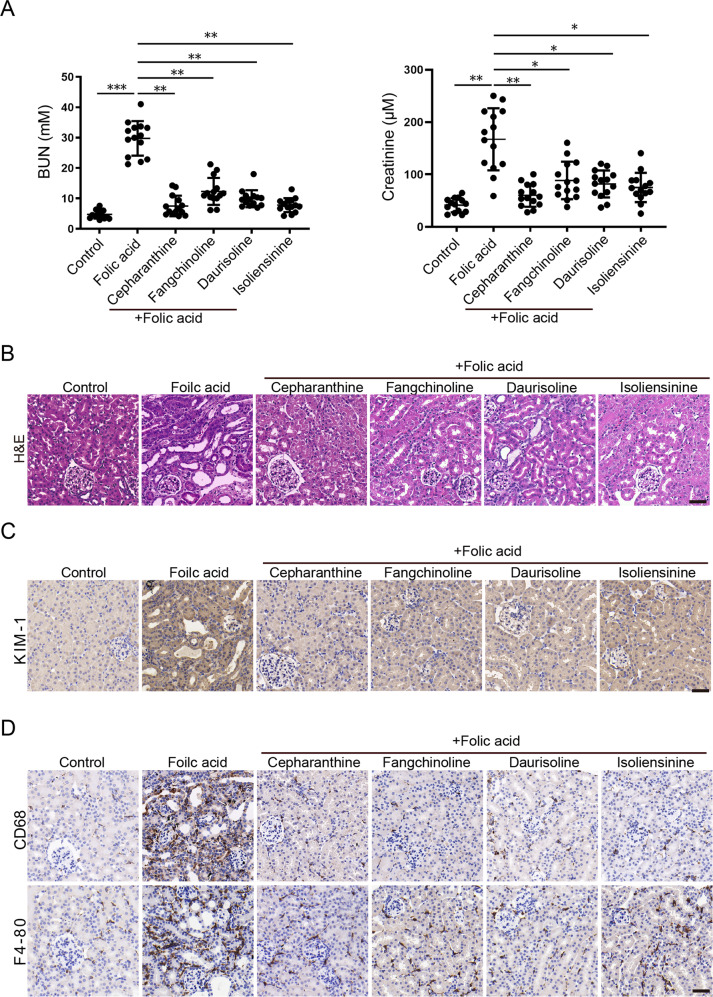
BBIQs protect mice from folic acid-induced acute kidney injury. C57BL/6J mice were randomly grouped (7 animals per group) and pretreated with the indicated testing compounds (*po*, 20 mg/kg) then treated with folic acid (*ip*, 250 mg/kg) for 48 h. Mice were then sacrificed and samples prepared for further analysis. Assays were repeated once. **A** Assessment of renal function in all treated mice by levels of blood urea nitrogen (BUN) and plasma creatinine. Data represent mean ± s.d. of 14 animals from two independent repeats and *p* value (vs. folic acid alone) is determined by ordinary one-way ANOVA with Dunnett’s multiple comparisons test. ns, no significance; *, *p* < 0.05; **, *p* < 0.01; ***, *p* < 0.001. **B** Representative H&E staining images showing kidney tubular injuries in folic acid-treated mice which is ameliorated by administration of BBIQs. **C** Representative immunohistochemistry staining images for kidney injury marker KIM-1 in samples analyzed in (**B**). **D** Immunohistochemistry analysis for the infiltration of CD68 or F4-80 positive monocytes/macrophages in samples as in (**B**). Scale bars in (**B**–**D**): 50 µm.

A variety of in vivo activities have been documented in literatures for BBIQ compounds, ranging from antiarrhythmic, antioxidants, antitumor, anti-inflammation, to protective activities against tissue damages induced by ischemia/reperfusion injury, chemotherapeutic reagents, etc. Our revelation of BBIQs as ferroptosis inhibitors indicate that some of their abovementioned functions might be attributed to their anti-ferroptotic activities in vivo. To evaluate whether BBIQs can be effectively used as ferroptosis inhibitors in vivo, we evaluated their cyto-protective activities in the nephrotoxic folic acid-induced acute kidney injury model where ferroptosis is the primary cause of tissue damage [12]. Cepharanthine, fangchinoline, daurisoline, and isoliensinine were chosen for this in vivo experiment because of their high in vitro anti-ferroptosis activity (cepharanthine and fangchinoline, Fig. 1C) or relative low cytotoxicity (daurisoline and isoliensinine, Supplementary Fig. 1A). Mice were pretreated intragastrically with cepharanthine, fangchinoline, daurisoline, or isoliensinine (all at 20 mg/kg) then injected intraperitoneally with a single dose of folic acid (250 mg/kg) and mice were analyzed 48 h after folic acid treatment. We first compared the levels of blood urea nitrogen (BUN) and plasma creatinine between control and treatment groups and found that the four BBIQs tested all reduce the BUN and plasma creatinine levels in folic acid-treated animals (Fig. 6A). Furthermore, the folic acid-induced expression of acute kidney injury marker *ngal* is also blocked by these BBIQs as determined by qRT-PCR (Supplementary Fig. 4A), indicating reduced renal injury and improved renal function in BBIQ-treated animals. We then performed histological analysis of kidney tissue in these animals and found the folic acid-induced renal damages is also reduced in BBIQ-rescued groups (Fig. 6B). Immunohistochemistry staining of kidney injury molecule-1 (KIM-1), an early biomarker of nephrotoxicity, further confirmed that BBIQs ameliorate the folic acid-induced AKI (Fig. 6C). Furthermore, we observed folic acid-induced renal infiltration of CD68 or F4-80 positive monocytes/macrophages is also inhibited by BBIQ treatment (Fig. 6D), indicating reduced inflammation in BBIQ-treated animals. We then analyzed the expression levels of several pro-inflammatory cytokines/chemokines by qRT-PCR and found that BBIQs effectively blocked the folic acid-induced renal expression of *mcp-1*, *il-6,* and *tnf-α* (Supplementary Fig. 4B), which further supports that BBIQ treatment ameliorates folic acid-induced renal inflammation and tissue damage.

**Fig. 7:**
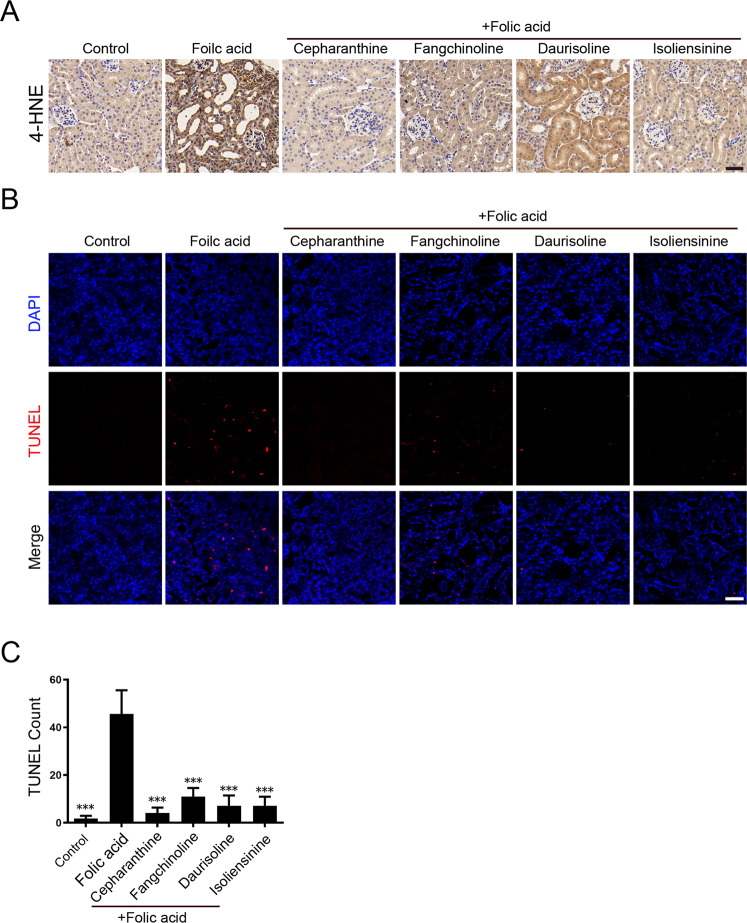
BBIQs reduce lipid peroxidation byproduct 4-HNE and suppress folic acid-induced renal cell death. **A** Representative immunohistochemistry staining results for 4-HNE, which is a byproduct of lipid peroxidation and a marker for oxidative stress. Scale bar: 50 µm. **B** Representative images of TUNEL assay to assess the level of cell death in renal sections. Scale bar: 50 µm. **C** Quantitative results for (**B**). Data represent mean ± s.d. of (TUNEL-positive count per 1000 cells) in sections from 7 mice for each treatment and *p* value (vs. folic acid alone) is determined by ordinary one-way ANOVA with Dunnett’s multiple comparisons test. ***, *p* < 0.001.

Our in vitro discovery that BBIQs inhibit ferroptosis by preventing the accumulation of detrimental lipid ROS indicates that similar mechanism could underlie renal protective function of BBIQs in FA-AKI. To test this possibility, we performed immunohistochemistry staining of 4-hydroxynonenal (4-HNE), a byproduct of lipid peroxidation, and revealed that all BBIQs reduce the formation of folic acid-induced 4-HNE in renal sections (Fig. 7A). We then analyzed cell death levels in the same samples by TUNEL assay and found the folic acid-induced cell death is also blocked by BBIQ treatment (Fig. 7B, C). Together, these date indicate that BBIQs are able to suppress accumulation of lipid ROS, reduce renal cell death and damage-induced inflammation and improve renal function in folic acid-treated mice.

## Discussion

Several BBIQ alkaloids identified as novel ferroptosis inhibitors in this study have been extensively investigated in previous studies. A variety of functions (sometimes contradictory) from cyto-protective activities (such as antioxidant activity, anti-inflammation, and immune-modulation, etc.) to anticancer activities (anti-proliferation and metastasis, antidrug resistance, induction of cell death, etc) have been attributed to them [19–23]. As long as ferroptosis is concerned, cepharanthine, dauricine, and neferine are recently reported to be involved in the regulation of ferroptosis in certain cells. Cepharanthine inhibits RSL3-induced ferroptosis in endothelial cells and attenuate brain injury after subarachnoid hemorrhage (SAH), presumably through downregulation of ALOX15 which is a positive regulator of ferroptosis [24]. However, ALOX15 has been shown to be dispensable for ferroptosis induced by GPX4-depletion in fibroblasts, T-cells, or renal tissues [10, 25] and a prevailing role of ALOX15 in ferroptosis is debatable [26]. Thus, it remains to be investigated whether cepharanthine can protect cells from ferroptosis in an ALOX15-independent manner. Meanwhile, dauricine protects ferroptotic brain damage after intracerebral hemorrhage through upregulating GPX4 and glutathione reductase (GSR) [27]. On the other hand, neferine which is another BBIQ compound we investigated in this study, is reported to promote apoptosis and ferroptosis in thyroid cancer cell lines through downregulation of NRF2 pathway [28]. We reported here that, in the well-documented RSL3-induced ferroptosis assay in HT1080 cells, BBIQs including cepharanthine, dauricine, and neferine do not affect protein levels of ferroptosis regulators including ALOX15, GPX4, or NRF2 (also known as NFE2L2). Instead, they can directly function as RTAs to scavenge lipid ROS. This observation suggests BBIQs to be protective against all ferroptotic stimuli. Thus, we propose that the lipid ROS scavenging activities of cepharanthine and dauricine contribute to their reported protective roles in hemorrhage-induced brain injury and other BBIQs we identified here are expected to have similar protective activity as well.

BBIQs have been reported to modulate ROS species in various cell types or cell-free systems either positively or negatively. For example, cell-free studies indicate cepharanthine can directly scavenge hydroxyl radicals or DPPH and inhibit lipid peroxidation in mitochondria initiated by Fe^2+^/ADP [29, 30]. Meanwhile, studies in certain cancer cell lines reported that cepharanthine can stimulate ROS generation and promote cell death in doxorubicin-resistant murine leukemia cells [31], human hepatocellular carcinoma cells [32], non-small-cell lung cancer cells [33], or glioma and neuronal cells [34]. Thus, the ROS scavenging activity in cell-free systems does not always predict a ROS-reducing effect in living cells. In this study, we found that cepharanthine scavenges DPPH (but not hydroxyl radical) and inhibits RSL3 or erastin-induced lipid peroxidation in HT1080 cells. Can ROS scavenging activity in DPPH assay be used to predict anti-ferroptosis capacity? A recent study revealed that many compounds with high radical scavenging activity in cell-free DPPH assay do not inhibit ferroptosis in cells [35], possible due to factors such as the relatively high compound dose used in cell-free assays which is likely to induce artificial effect, the radical species and their distribution in cell-free system are very different from those critical for ferroptosis, cell-free assay cannot evaluate the ability of a compound to partition into lipid environments in living cells, etc. Thus, the potential anti-ferroptosis capacity of a ROS scavenger need to be directly evaluated in well-defined and well-controlled experimental systems.

Although the reported functions of BBIQ alkaloids in cancer cells or other in vitro cultured cells are often contradictory, a general theme from in vivo studies is that BBIQs protect tissue damages under a variety of pathogenic conditions. For example, cepharanthine have been reported to protect renal injuries induced by ischemia/reperfusion [36, 37], chemotherapeutic reagent cisplatin [38], streptozotocin-induced diabetic nephropathy [39], transient middle cerebral artery occlusion (tMCAO) or subarachnoid hemorrhage induced brain injury [24, 40] or dextran sulfate sodium (DSS)-induced ulcerative colitis [41]. Protective functions of fangchinoline have been reported in models of oxidative glutamate toxicity [42], streptozotocin-induced diabetic nephropathy [43] as well as or LPS-induced cardiac dysfunction [44]. In addition to its well-studied anti-hypertension and anti-arrhythmia function [45], berbamine is reported to protect ischemia/reperfusion-induced heart injury [46, 47] and ethanol-induced liver damage [48]. Neferine is reported to attenuate ischemia/reperfusion or LPS-induced AKI [49], cerebral ischemia-induced brain damage [50], and DSS-induced ulcerative colitis [51]. Liensinine is reported to ameliorate doxorubicin-induced cardiomyopathy [52] and isoliensinine inhibits bleomycin-induced pulmonary fibrosis [53]. Although a variety of mechanisms such as anti-oxidation, immune-modulation, or modulation of gut microbiota have been proposed, direct targets and prevailing pathways responsible for the cyto-protective functions of BBIQs in these disease models remain to be established. In light of recent discoveries about the involvement of ferroptosis in an expanding list of pathological conditions [26, 54, 55], our discovery of BBIQs as ferroptosis inhibitors provide novel opportunities to investigate tissue protective mechanisms of BBIQs in these disease models.

The coronavirus disease 2019 (COVID-19) pandemic caused by the infection of severe acute respiratory syndrome coronavirus 2 (SARS-CoV-2) promotes the identification and development of small chemical compounds with antiviral activities. In vitro cell culture-based SARS-CoV-2 infection assays have been used by many research laboratories to screen a variety of compound libraries and several BBIQs were repeatedly identified as antiviral candidate compounds. For example, cepharanthine and berbamine were identified as potential anti-SARS-CoV-2 agents by several groups [56–58]. Further studies reported that cepharanthine blocks SARS-CoV-2 entry into target cells [59, 60] while berbamine inhibits SAR-CoV-2 infection by interfering endolysosomal trafficking of ACE2 [61] or blocking the S protein-mediated membrane fusion [62]. Neferine suppresses SARS-CoV-2 infection by inhibiting Ca^2+^-dependent membrane fusion and virus entry [63]. Daurisoline inhibits replication of SARS-CoV-2 in host cells possibly through an autophagy-related mechanism [64]. These studies revealed a conserved antiviral activity of these BBIQs while the proposed antiviral mechanism for each compound is largely based on previously reported activity of the compound. Our finding that these BBIQs have a common mechanism as RTAs to restrict lipid peroxidation and block ferroptosis indicates that BBIQs might have additional function and mechanism during virus infection, especially in light of possible ferroptotic tissue damages induced by SARS-CoV-2 infection as reported in recent studies [65–67].

In summary, our study identified a group of in vivo applicable ferroptosis inhibitors which may facilitate in vivo ferroptosis studies and provide novel opportunity to protect ferroptotic tissue damages under a variety of pathological conditions.

## Materials and methods

### Compounds

Alkaloid natural product library was obtained from TargetMol (TargetMol, Shanghai, China, L6110). RSL3 (Selleckchem, Houston, TX, S8155) and erastin (Selleckchem, S7242) were used to induce ferroptosis. Staurosporine (Selleckchem, S1421), rapamycin (Selleckchem, S1039), rotenone (Selleckchem, S2348)/H_2_O_2_ (Sigma-Aldrich, MO, 31642) were used to induce apoptosis, autophagy, and necrosis, respectively. Ferrostatin-1 (Selleckchem, S7243)/liproxstatin-1 (Selleckchem, S7699), Z-VAD-FMK (Selleckchem, S8102), 3-methyladenine (Selleckchem, S2767), necrostatin-1 (Selleckchem, S8037) were used to inhibit ferroptosis, apoptosis, autophagic cell death and necrosis, respectively. N-acetylcysteine (NAC) (Selleckchem, S1623) is an antioxidant that removes ROS. BBIQ compounds used in the study were all obtained from Selleckchem which include fangchinoline (S3611), cepharanthine (S4238), berbamine (S9141), dauricine (S9295), daurisoline (S9150), neferine (S5144), isoliensinine (S9247), liensinine (S9411).

### Cell culture

HT1080 (ATCC, CCL-121), MDA-MB-231 (ATCC, CRM-HTB-26), and H1975 (ATCC, MD, CRL-5908) cells were purchased from ATCC. HepG2 and U251 cells were from stocks maintained in our lab. Cells were routinely tested for mycoplasma contamination. HT1080, U251, and HepG2 cells were cultured in DMEM High Glucose cell culture medium (HyClone, UT, SH3022.01) supplemented with 10% fetal bovine serum (NTC, Argentina, SFBE) and 1% Penicillin-Streptomycin Solution (HyClone, SV30010). MDA-MB-231 and H1975 cells were cultured in RPMI 1640 cell culture medium (Gibico, CA, C11875500BT) supplemented with 10% fetal bovine serum (NTC, SFBE) and 1% Penicillin-Streptomycin Solution (HyClone, SV30010). Cells were all cultured at 37 °C with 5% CO_2_ (Thermo, MA, HERACELL150i).

### Compound treatment

For alkaloid natural product library screen, HT1080 cells in 80% confluency were trysinized to single cell suspension, seeded on 96-well plates at the density of 8000 cells per well for 24 h, then treated with RSL3 (2 μM) plus a testing compound (5 μM) diluted in culture media. Cell viability was determined 24 h after treatment by CCK-8 assay as described below. Assays were performed in triplicates and repeated three times. The top 20 hits from the screen were further tested for their abilities to protect HT1080 cells from cell death induced additional stimuli which included erastin (5 μM), staurosporine (1 μM), rapamycin (10 μM), rotenone (3 μM) or H_2_O_2_ (50 μM). Ferrostatin-1/liproxstatin-1 (5 μM), NAC (1 mM), Z-VAD-FMK (50 μM), 3-methyladenine (5 mM), and necrostatin-1 (50 μM) were used as control. For the eight BBIQ compounds in the top 20 hits, dose responses in range of 0.5 to 30 μM were assayed in HT1080 cells and their anti-ferroptotic activity were further tested in additional cell lines (H1975, U251, MDA-MB-231, and HepG2). Cells cultured in their own growth media were treated and assayed using the same protocol described above.

### Cell viability assay

Cell viability was assayed using the Cell Counting Kit-8 (CCK-8) (Biosharp, Beijing, China, BS350B) with manufacturer’s protocol. Briefly, the CCK-8 stock solution was diluted (1:10) in complete cell culture medium to make the working solution. At the end of compound treatment, cell culture medium was removed and replaced with 110 μl of CCK-8 working solution and cells were cultured at 37 °C in dark for 1 h then put into an automatic microplate reader (Thermo, Epoch 2) to measure OD_450_. Cell viability was then calculated as following: Cell Viability = [(Test Group OD_450 _− Blank group OD_450_)/(Control Group OD_450_ − Blank Group OD_450_)] × 100%. All assays were performed in triplicates and repeated at least three times.

### Flow cytometry analysis of ROS

HT1080 cells were seeded on 12-well plates at the density of 15,000 cells per well overnight then treated with RSL3 (2 μM) or rotenone (2 μM) in the presence or absence of ferrostatin-1 (5 μM), NAC (5 mM), or a testing BBIQ compound (5 μM). Cells were treated for 4 h at 37 °C, then washed 3 times with FBS-free DMEM cell and ready for fluorescent dye labeling. For detection of cytosolic ROS, DCFH-DA probe (Beyotime, Shanghai, China, S0033S) was diluted in serum-free medium to final concentration of 10 μM. Cells at the end of drug treatment were incubated in DCFH-DA working solution for 30 min at 37 ˚C in dark. For detection of lipid ROS, cells were incubated in the C11 BODIPY 581/591 lipid ROS probe (Invitrogen, NY, D3861) diluted in serum-free media at the final concentration of 2 μM for 20 min in dark. For detection of mitochondrial ROS, cells were stained in 5 μM of MitoSOX Red Mitochondrial Superoxide Indicator (Invitrogen, M36008) for 10 min in dark. After dye incubation, cells were washed 3 times with serum-free cell culture medium, trypsinized with 100 μl 0.25% Trypsin-EDTA (Gibico, 25200056). Trypsinization was terminated by adding 1 ml of complete DMEM culture medium and cells were centrifuged at 1300 rpm for 3 min and then resuspended in sterile PBS containing 5% FBS. Cell suspension was filtrated by a 70-μm filter, and then analyzed using the BD Accuri C6 Plus Flow Cytometer (BD, NJ) and the BD FlowJo V10 software.

### Immunofluorescence staining of ROS

HT1080 cells were seeded on coverslip (WPI, FL, FD35–100) at the density of 150,000 cells per 30-mm dish or 24 h. Cells were then treated and stained with probes for various ROS species as described above. After ROS labeling, cells were washed then incubated in Hoechst 33258 (1:1000 diluted in serum-free DMEM medium) for 5 min. Cells were then washed with DMEM medium and imaged using the LSM800 fluorescence confocal microscope (Zeiss, Germany).

### Western blotting

Cells cultured in 30-mm dish were treated with the indicated compound as described above. After compound treatment, 300 µl RIPA (Thermo, 89901) containing 1% PMSF (Yeasen, Shanghai, China, 20104ES03) and 1% protease inhibitor cocktail (Yeasen, 20123ES50) was added to cell culture and samples were put on ice for 10 min. The mixture was then centrifuged at 12,000 pm for 10 min at 4 °C. The supernatant was transferred to a new EP tube, mixed thoroughly with 1/4 volume of the loading dye (Beyotine, P0015), and incubated in boiling water for 15 min. Samples were centrifuged at 13,000 rpm for 3 min and supernatants were separated by 12% SDS-PAGE, transferred to PVDF membranes (EMD Millipore, MA, IPVH00010). Membranes were blocked in solution (5% skim milk in TBST) for 1 h, washed with TBST for 3 times, then incubated with a primary antibody diluted in QuickBlock™ Primary Antibody Dilution Buffer for Western Blot (Beyotime, P0256) overnight at 4 °C. Blots were then washed with TBST and incubated with a HRP-conjugated secondary antibodies (Goat Anti-Mouse IgG (H + L) (Proteitech, Wuhan, China, SA00001-1) or Goat Anti-Rabbit IgG (H + L) (Proteitech, SA00001-2)) for at RT for 1 h. Blots were then washed with TBST extensively and detected using the Immobilon Western Chemiluminescent HRP Substrate (EMD Millipore, WBKLS0500). The primary antibodies used in this study were: anti-GPX4 (1:1000, Abcam, Cambridge, UK, ab125066), anti-SLC7A11 (1:1000, Proteintech, 26864-1), anti-ACSL4 (1:1000, Abcam, ab205199), anti-FTH1 (1:1000, Abcam, ab75973), anti-TFRC (1:1000, Abcam, ab269513), anti-ALOX15 (1:1000, GeneTex, CA, GTX33001), anti-FSP1 (1:1000, Affinity, OH, DF8636), anti-TP53 (1:1000, Affinity, BF8013), anti-NFE2L2 (1:1000, Abcam, ab62352), anti-GAPDH (1:1000, Proteintech, HRP-60004).

### DPPH assay

The free radical scavenging capacity of BBIQ compounds was assayed using the DPPH Assay Kit (Solarbio, Beijing, China, BC4755). Briefly, testing compounds were diluted in the provided solution to the concentration of 1, 5, 10, or 50 µM. An aliquot of testing compound solution (10 µl) was mixed thoroughly with 190 µl of DPPH working solution in 96-well plate and incubated at RT in dark for 30 min. After reaction, the absorbance (A) of the reaction mixture at the wave length of 515 nm was assayed using an automatic microplate reader (Thermo, Epoch 2). The DPPH scavenging rate is calculated by the formula: positive control: DVC% = [(A blank − A positive control)/A blank] × 100%; sample: D% = [[A blank − (A sample − A control)]/A blank] × 100%.

### Hydroxyl radical assay

The hydroxyl radical scavenging capacity of a compound was assayed with the hydroxyl radical scavenging ability detection Kit (Solarbio, BC1320). Briefly, a testing compound was diluted in the provided solution to the final concentration of 50 µM. NAC (5 mM) was used as positive control. Compound solution was mixed with the hydroxyl radical working solution and incubated in 37 °C water bath for 60 min. Samples were then centrifuged at 10,000 rpm at RT for 10 min, supernatant was taken to measure absorbance at 536 nm. The hydroxyl radical scavenging rate is calculated by the formula: D% = (A sample − A control)/(A blank − A control) × 100%.

### Folic acid-induced acute kidney injury (FA-AKI) model

Animal procedures were approved by the Institutional Animal Care and Use Committee of Guangzhou Institutes of Biomedicine and Health, Chinese Academy of Science (Issue No. 2021034). Ferroptosis is the primary cause of folic acid-induced acute kidney injury so we used this protocol to evaluate in vivo anti-ferroptosis activity of BBIQs. C57BL/6J mice (8–10 weeks old) were purchased from Vital River Laboratory Animal Technology Company (Zhejiang, China) and were adapted to the housing conditions for one week before compound treatment. To induce AKI, mice (7 per experimental group) received a single intraperitoneal injection of FA (Sigma-Aldrich, F7876) dissolved in 0.3 M sodium bicarbonate at the dosage of 250 mg/kg. The control group was injected with vehicle (0.3 M sodium bicarbonate, 200 µl). BBIQs generally have limited solubility and bioavailability in vivo, so mice were pretreated with BBIQs before FA treatment. Specifically, cepharanthine, fangchinoline, daurisoline, or isoliensinine were firstly dissolved in DMSO (100 mg/ml) then diluted to 1.25 mg/ml in 0.5% CMC-Na (Sigma, 9004-32-4). Mice were dosed intragastrically with cepharanthine, fangchinoline, daurisoline, or isoliensinine (all at 20 mg/kg, based on results from our preliminary experiments) daily from 2-day prior to FA injection to 1-day after that (48, 24, 2 h before FA treatment plus 24 h after FA treatment). Mice were then euthanized 48 h after FA injection. Plasma samples were collected at the time of euthanasia. Kidneys were perfused in situ with cold saline before removal and one kidney was frozen in liquid nitrogen for RNA preparation and the other was perfused with cold 4% PFA and then fixed for paraffin or frozen section.

### Renal function assessment

Renal function was assessed by measuring the plasma creatinine and blood urea nitrogen (BUN) levels, two routinely used parameters for detecting acute kidney injury. The blood samples collected in heparin sodium-coated blood collection tube were centrifuged at 1000 rpm for 10 min then the plasma was transferred to new 1.5 ml EP tube. Plasma creatinine levels were assayed using the Creatinine Assay Kit (Nanjing Jiancheng Bioengineering Institute, C011-2-1). Plasma BUN levels were assayed with the Urea Assay Kit (Nanjing Jiancheng Bioengineering Institute, Nanjing, China, C013-1-1) according to the manufacturer’s instructions.

### H&E and immunohistochemistry staining

For H&E staining, kidneys fixed in 4% PFA were embedded in paraffin then sectioned at the thickness of 5 µm. Haematoxylin and eosin (H&E) staining of sections was performed using the protocol provided by the manufacturer (Beyotime, C0105M). For immunohistochemistry, paraffin sections were fixed in pre-cooled acetone at 4 °C for 10 min then washed with PBS. After three washes, sections were incubated in 3% H_2_O_2_/methanol for 20 min at RT, followed by PBS wash (twice, 5 min each). Sections were blocked in 5% BSA/PBS for 10 min at RT then incubated with a primary antibody (anti-KIM-1 (1:100, Immunoway, TX, YT5888), anti-F4/80 (1:100, CST, MA, 70076S), anti-CD68 (1:100, CST, 97778S), anti-4-HNE (1:100, Abcam, ab48506)) overnight at 4 °C, and then followed by secondary antibody incubation (30 min at 37 °C). Three times of PBS wash were performed before and after incubation of secondary antibody. Finally, sections were incubated with the DAB solution (Beyotime, P0202) in dark for 10 min at RT, washed with tap water, and then counterstained with haematoxylin (Beyotime, C0107). Sections were then examined and photographed with the LSM 800 fluorescence confocal microscope (Zeiss).

### RNA extraction and qRT-PCR

The frozen kidney tissues were firstly homogenized in 500 µl cold saline and the FastPure Cell/Tissue Total RNA Isolation Kit V2 (Vazyme, RC112) was used for RNA extraction according to manufacturer’s instructions. Reverse transcription of RNA was performed using the HiScript III RT SuperMix for qPCR (+gDNA wiper) (Vazyme, R323). qPCR was performed using the CFX96 Touch Real-Time PCR Detection System (BioRad, CA). Primers used in this study were: GAPDH, 5′-ATCATCCCTGCATCCACT-3′ and 5′-ATCCACGACGGACACATT-3′; NGAL, 5′-AATGTCACCTCCATCCTGGT-3′ and 5′-ATTTCCCAGAGTGAACTGGC-3′; TNF-α, 5′-CAGGCGGTGCCTATGTCTC-3′ and 5′-CGATCACCCCGAAGTTCAGTAG-3′; MCP-1, 5′-CCTGCTGTTCACAGTTGCC-3′ and 5′-ATTGGGATCATCTTGCTGGT-3′; IL-6, 5′-AGTCCGGAGAGGAGACTTCA-3′ and 5′-ATTTCCACGATTTCCCAGAG-3′.

### TUNEL assay

Frozen sections were prepared from kidneys fixed in 4% PFA using the Leica CM3050S Microtome. Sections were fixed with 4% PFA again for 30 min followed by 2 PBS wash. Sections were incubated with 0.5% Triton X-100/PBS for 5 min then incubated in TUNEL solution (Beyotime, C1090) in dark at 37 °C for 60 min. Sections were counterstained with DAPI (1:5000 in PBS, Beyotime, C1005) for 5 min, washed then imaged using the LSM800 fluorescence confocal microscope (Zeiss). At least three sections from three different mice were analyzed for each treatment.

### Statistical analyses

Statistical analyses were performed using the GraphPad Prism 8.0.2 software (GraphPad Software, CA). Data were collected from 3 independent biological repeats and expressed as means ± s.d. Statistical significance was determined using one-way or two-way ANOVA, and *p* values <0.05 were considered statistically significant. Sample sizes subjected to statistical analysis were at least 7 animals per group.

## Supplementary information

Supplementary Figures

Supplementary Table 1

Original western blots

aj-checklist

## Data Availability

The data in the article and its supplementary information files are available from the authors on reasonable request.
