# Supplementary material for: Identification of a group of bisbenzylisoquinoline (BBIQ) compounds as ferroptosis inhibitors
Source: Cell Death Dis. 2022 Nov 26;13(11):1000. doi: 10.1038/s41419-022-05447-8 (PMC9701226; doi:10.1038/s41419-022-05447-8)
Supplement: Supplementary file 1 — Supplementary Figures [file 41419_2022_5447_MOESM1_ESM.docx]

**
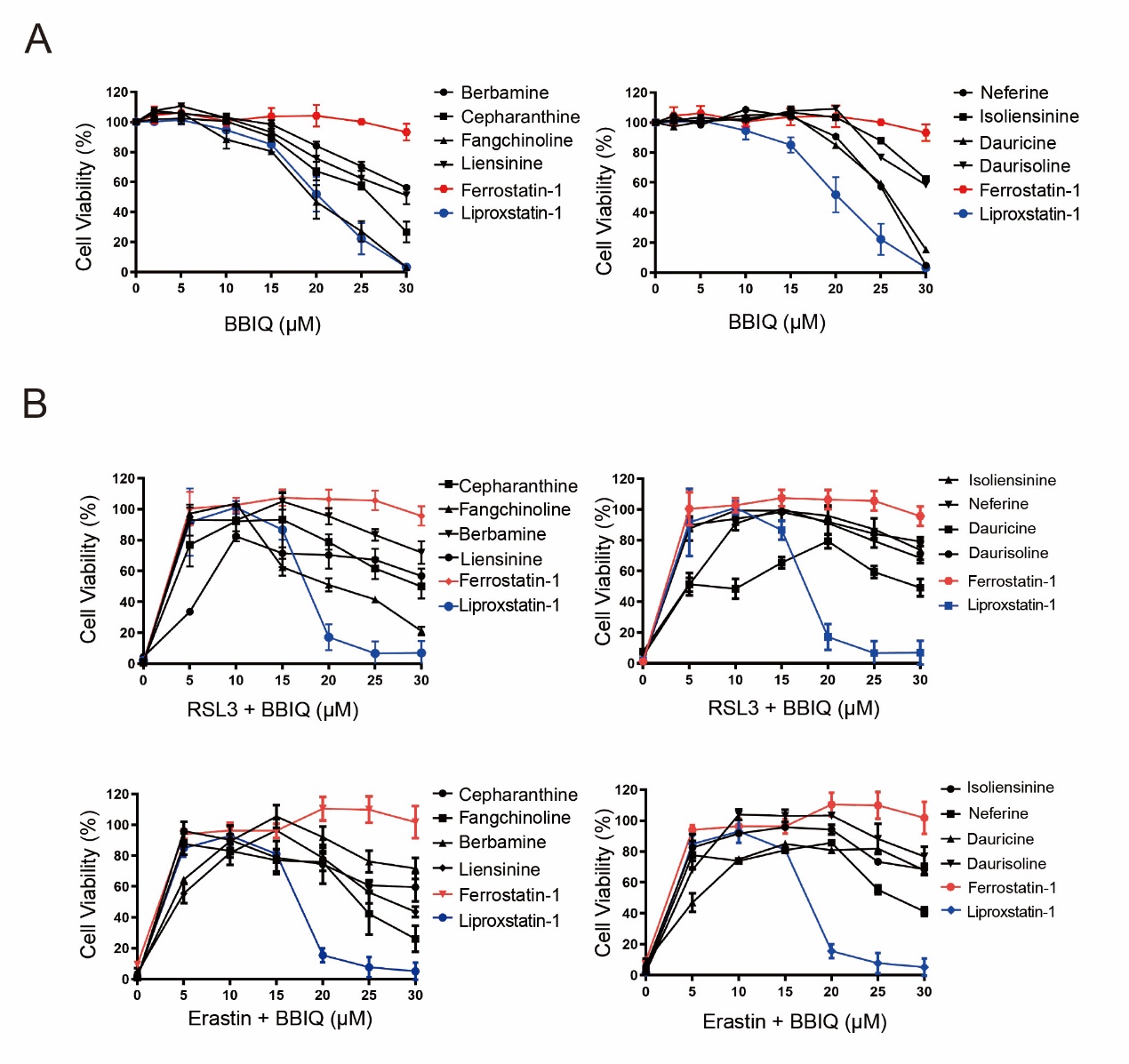
**

**Supplementary Figure 1. Dose-response of BBIQs in HT1080 cells. A** Potential cytotoxicity of BBIQ compounds. Cells were treated with the indicated concentrations of ferrostatin-1, liproxstatin-1 or the indicated BBIQ compound for 24 h then cell viability determined by CCK-8 kit as in Figure 1c. **B** Dose response curves for BBIQ compounds in rescuing RSL3 (2 µM) or erastin (5 µM) induced ferroptosis in HT1080 cells. Data represent mean ± s.d. from three independent experiments.

**
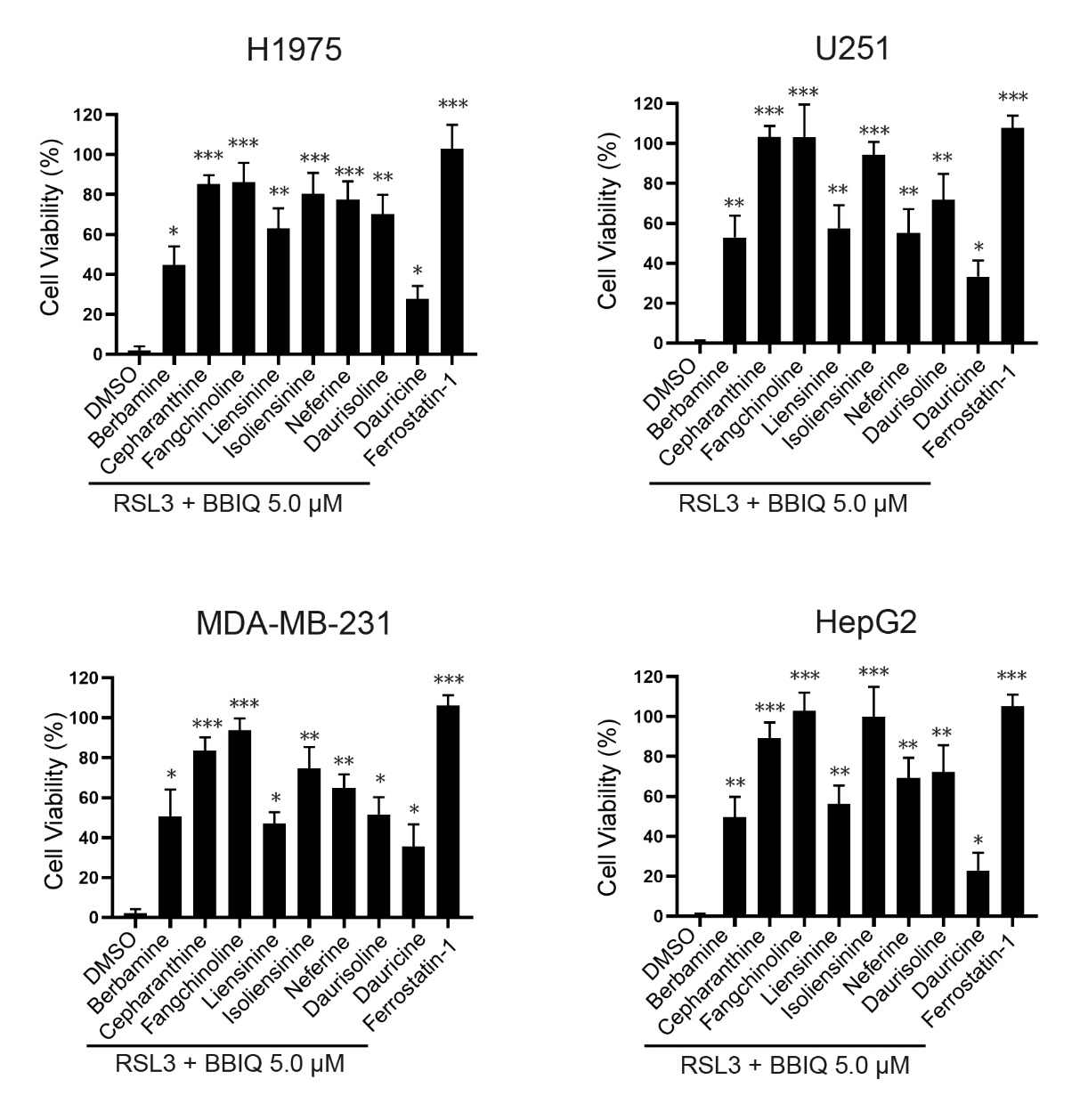
**

**Supplementary Figure 2. BBIQ compounds inhibit RSL3-induced ferroptosis in multiple cell lines.** Cells were treated with RSL3 (2 μM) plus a testing compound (5 μM) or ferrostatin-1 (5 μM) for 24 h then cell viability determined. Data represent mean ± s.d. from three independent experiments and *p* value (vs. DMSO) is determined by ordinary one-way ANOVA with Dunnett’s multiple comparisons test. *, *p* < 0.05; **, *p* < 0.01; ***, *p* < 0.001.

**
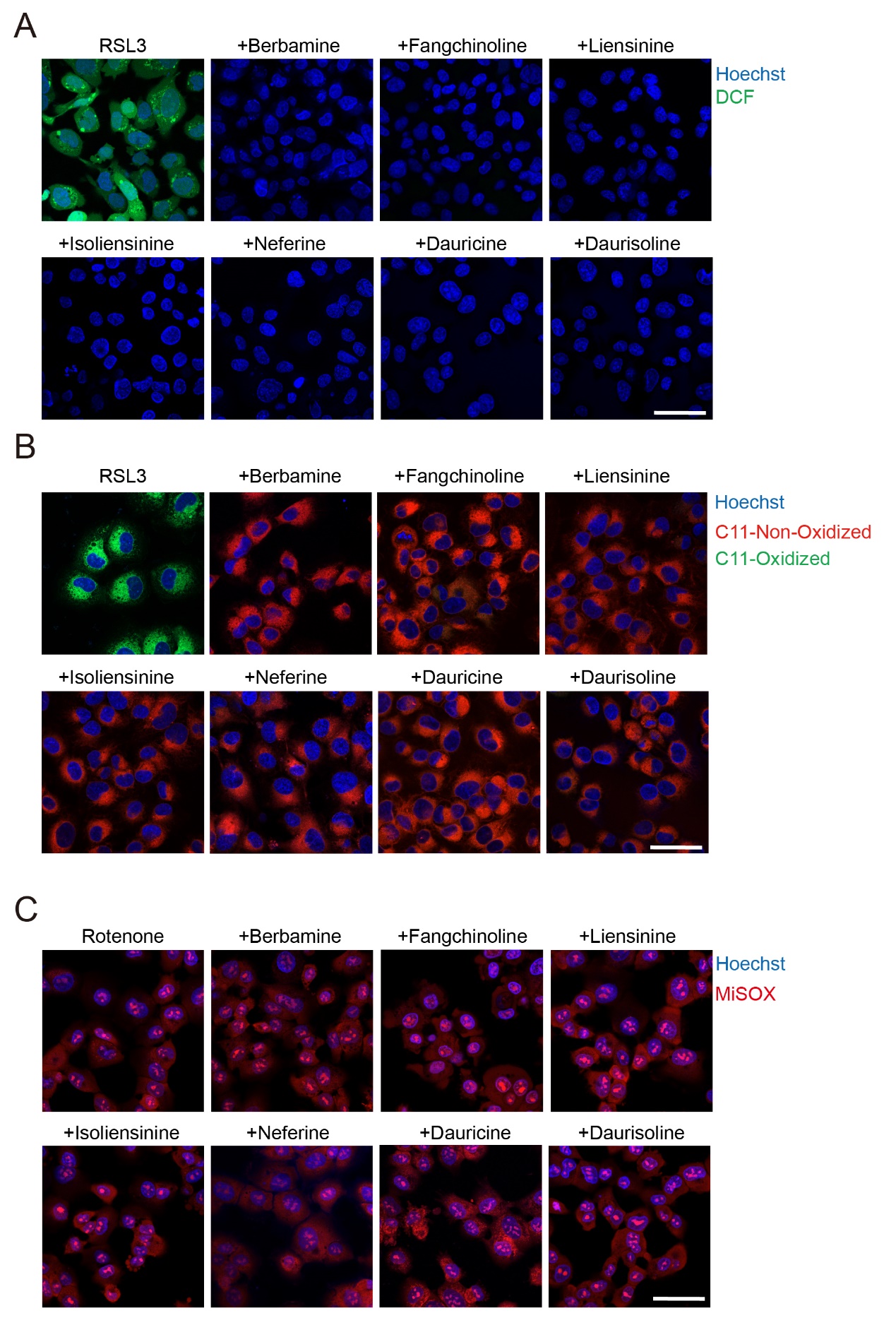
**

**Supplementary Figure 3. Immunofluorescence staining of various ROS species in HT1080 cells. A** Representative images of RSL3-induced cytosolic ROS by DCF staining. Assays were performed as in Figure 2A. **B** Representative C11-BODIPY staining of lipid ROS induced by RSL3. Assays were performed as in Figure 3A. **C** Representative MitoSOX staining of mitochondrial ROS induced by rotenone. Assays were performed as in Figure 4A. Scale bars in A-C: 50 μm.


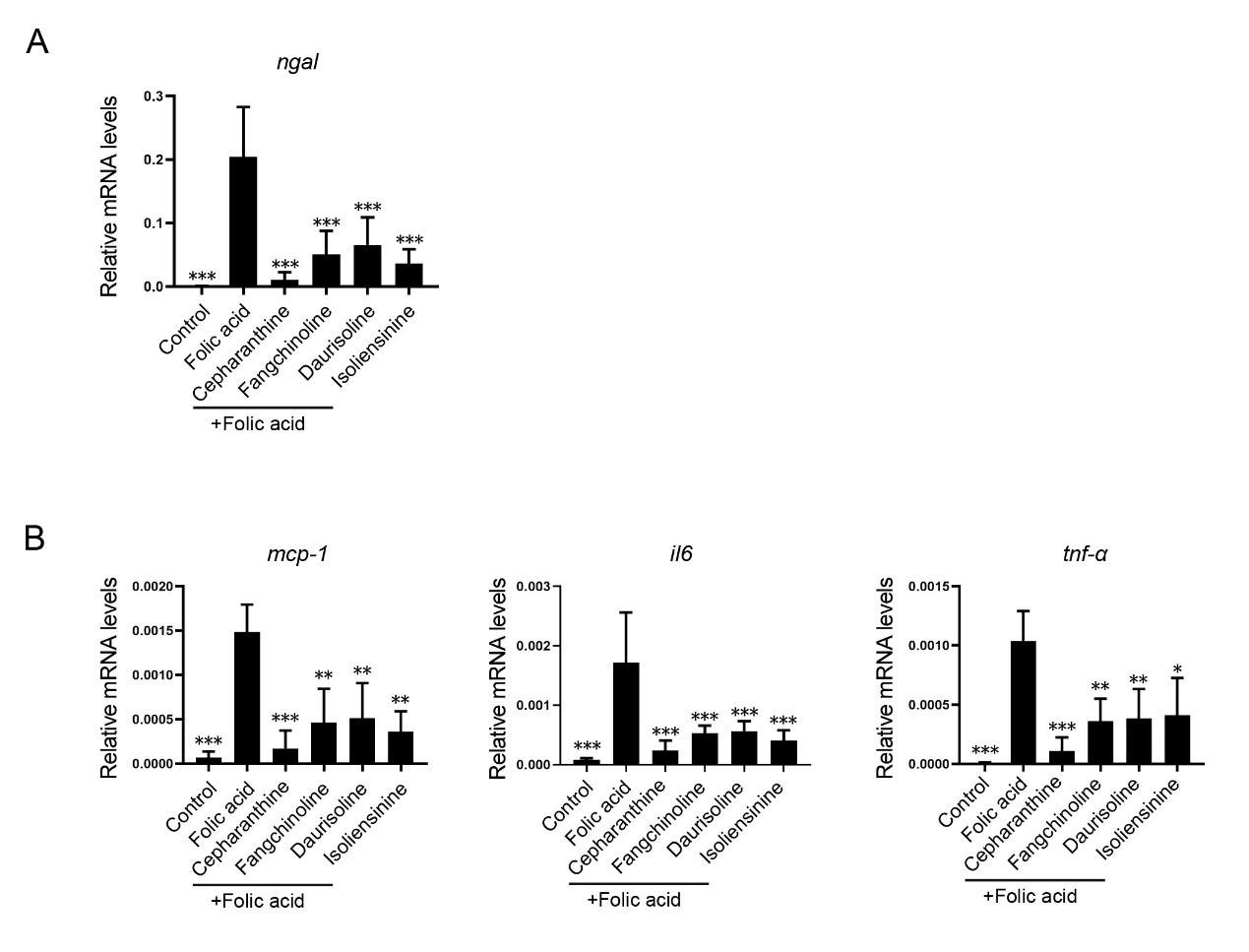


**Supplementary Figure 4. BBIQs protect mice from folic acid-induced acute kidney injury. A** qRT-PCR analysis of acute renal injury marker *ngal* in kidney samples. **B** qRT-PCR analysis for the expression of inflammatory cytokine genes in the same samples as analyzed in A. Mice were treated as described in Figure 6. *gapdh* is the internal control. Data represent mean ± s.d. and *p* value (vs. folic acid alone) is determined by ordinary one-way ANOVA with Dunnett’s multiple comparisons test. *, *p* < 0.05; **, *p* < 0.01; ***, *p* < 0.001.
