# Supplementary material for: Identification of a group of bisbenzylisoquinoline (BBIQ) compounds as ferroptosis inhibitors
Source: Cell Death Dis. 2022 Nov 26;13(11):1000. doi: 10.1038/s41419-022-05447-8 (PMC9701226; doi:10.1038/s41419-022-05447-8)
Supplement: Supplementary file 2 — Supplementary Table 1 [file 41419_2022_5447_MOESM2_ESM.pdf]

Cat #	Compound Name	CAS #	Formula	Molecular Weight	(Cell Viability) % (RSL3+Compound)
T5S2360	Corydaline	518-69-4	C22H27NO4	369.46	108.23%
T4S0795	Berberrubine	15401-69-1	C19H16ClNO4	357.79	103.11%
T3054	Daurisoline	70553-76-3	C37H42N2O6	610.75	101.72%
T0131	Cepharanthine	481-49-2	C37H38N2O6	606.73	101.44%
T3369	Nuciferine	475-83-2	C19H21NO2	295.38	101.09%
T2874	Tetrahydroberberine	522-97-4	C20H21O4N	339.39	100.44%
T3246	Tabersonine	4429-63-4	C21H24N2O2	336.43	99.17%
T2809	(+)-Thalrugosine	33889-68-8	C37H40N2O6	608.72	98.89%
T5807	Stylopine hydrochloride	96087-21-7	C19H18ClNO4	359.8	98.56%
T2S2215	Crebanine	25127-29-1	C20H21NO4	339.39	98.15%
T8195	Lotusine	6871-67-6	C19H24NO3+	314.4	95.13%
T5S1099	Liensinine	2586-96-1	C37H42N2O6	610.75	93.44%
T3122	(+)-Fangchinoline	436-77-1	C37H40N2O6	608.72	92.90%
T5S1103	Isoliensinine	6817-41-0	C37H42N2O6	610.75	91.29%
T7786	Tryptanthrin	13220-57-0	C15H8N2O2	248.24	89.77%
T6S0119	Dauricine	524-17-4	C38H44N2O6	624.77	87.11%
T4S2063	Tetrahydrocoptisine	7461-02-1	C19H17NO4	323.34	86.68%
T5S1895	Norisoboldine	23599-69-1	C18H19NO4	313.35	86.22%
T5S1097	Neferine	2292-16-2	C38H44N2O6	624.77	86.02%
T3S0807	Berbamine	478-61-5	C37H40N2O6	608.73	85.55%
T8149	Dobutamine (hydrochloride)	49745-95-1	C18H24ClNO3	337.84	85.29%
T2782L	Catharanthine Tartrate(2468-2	T2782L	C25H30N2O8	486.51	85.07%
TN1091	Dehydronuciferine	7630-74-2	C19H19NO2	293.36	83.21%
T5720	Boldine	476-70-0	C19H21NO4	327.37	81.94%
T8187	Tetrahydroepiberberine	38853-67-7	C20H21NO4	339.4	81.31%
T2920	Berbamine dihydrochloride	6078-17-7	C37H42Cl2N2O6	680.24	79.56%
T0791	Reserpine	50-55-5	C33H40N2O9	608.68	77.74%
TN1969	N-(p-Coumaroyl) serotonin	68573-24-0	C19H18N2O3	322.4	75.66%
TN2252	Syrosingopine	84-36-6	C35H42N2O11	666.71	73.62%
TQ0296L	N-Nornuciferine hydrochloride	TQ0296L	C18H20ClNO2	317.81	63.93%
T3S0057	Dihydrochelerythrine	6880-91-7	C21H19NO4	349.38	62.38%
T4614	Ellipticine hydrochloride	5081-48-1	C17H15ClN2	282.76	61.47%
TN1032	Geissoschizine methyl ether	60314-89-8	C22H26N2O3	366.45	59.20%
T2714	Rotundine	10097-84-4	C21H25NO4	355.43	57.77%
T2S1792	Lycobetaine	72510-04-4	C16H12NO3+	266.27	57.61%

T4S0794	(R)-(+)-Corypalmine	13063-54-2	C20H23NO4	341.4	52.44%
T3152	Harmol	487-03-6	C12H10N2O	198.22	48.74%
T2832	S-Isocorydine(+)	475-67-2	C20H23NO4	341.41	47.89%
T4S2012	Dihydro Sanguinarine	3606-45-9	C20H15NO4	333.34	45.60%
T2917	Tetrahydropalmatine hydrochl	6024-85-7	C21H26ClNO4	391.89	45.49%
T5S0814	Berberine hydrogen sulphate	633-66-9	C20H19NO8S	433.43	38.14%
T4S1102	Liensinine diperchlorate	5088-90-4	C37H44Cl2N2O14	811.67	36.68%
T8189	Dihydroberberine	483-15-8	C20H19NO4	337.4	34.31%
T5S2357	Acetylcorynoline	18797-80-3	C23H23NO6	409.43	33.67%
T3S1457	Palmatrubine	16176-68-4	C20H20NO4+	338.38	32.10%
T3419	Chelerythrine chloride	3895-92-9	C21H18ClNO4	383.83	26.84%
T4S0779	D-tetrahydropalmatine	3520-14-7	C21H25NO4	355.4	25.44%
T8859	Nitidine	6872-57-7	C21H18NO4+	348.37	25.17%
T3933	Jatrorrhizine	3621-38-3	C20H20NO4	338.38	22.15%
T2996	Tetrandrine	518-34-3	C38H42N2O6	622.77	22.14%
T4S0797	Berberine	2086-83-1	C20H18NO4+	336.36	18.03%
T0461	Berberine hydrochloride	633-65-8	C20H18ClNO4	371.8	18.00%
T2793	Tetrahydropalmatine	2934-97-6	C21H25NO4	355.44	16.97%
T3013	Catharanthine tartrate	4168-17-6	C25H30N2O8	486.51	16.78%
T3S1852	Cephaeline hydrochloride	3738-70-3	C28H39ClN2O4	503.08	16.74%
T4429	Rauwolscine hydrochloride	6211-32-1	C21H27ClN2O3	390.9	16.48%
T2781	Sanguinarine	2447-54-3	C20H14NO4	332.33	15.91%
T3232	Higenamine Hydrochloride	11041-94-4	C16H17NO3·HCl	307.77	14.82%
T5S0056	Coptisine chloride	6020-18-4	C19H14ClNO4	355.77	14.72%
T2868	Evodiamine	518-17-2	C19H17N3O	303.35	14.71%
T1668	Vinblastine sulfate	143-67-9	C46H58N4O9·H2SO4	909.06	14.60%
TN1189	13-Methylberberine	54260-72-9	C21H20ClNO4	385.84	14.56%
T4S0800	Demethyleneberberine	25459-91-0	C19H18NO4+	324.35	14.46%
T8039	Brucine sulfate heptahydrate	60583-39-3	C46H68N4O19S	1013.1	14.36%
T5S2361	Epiberberine	6873-09-2	C20H18NO4+	336.36	14.30%
T8185	Viroallosecurinine	1857-30-3	C13H15NO2	217.26	14.23%
T5760	Sinoacutine	4090-18-0	C19H21NO4	327.37	14.15%
TN1142	8-Oxycoptisine	19716-61-1	C19H13NO5	335.31	14.11%
T1287	Synephrine	94-07-5	C9H13NO2	167.21	14.01%
T4036	Solasodine	126-17-0	C27H43NO2	413.65	13.95%
T7671	Myosmine	532-12-7	C9H10N2	146.19	13.86%
T1286	Vincamine	1617-90-9	C21H26N2O3	354.44	13.84%

T8182	Guan-fu base A	1394-48-5	C24H31NO6	429.5	13.80%
T5S0053	Coptisine	3486-66-6	C19H14NO4+	320.32	13.78%
T6213	Vinorelbine Tartrate	125317-39-7	C45H54N4O8·2C4H6O6	1079.11	13.67%
T1644	Dopamine hydrochloride	62-31-7	C8H12ClNO2	189.64	13.61%
T4S0051	Coptisine sulfate	1198398-71-8	C19H14NO4+	320.32	13.56%
T4592	Tomatidine hydrochloride	6192-62-7	C27H46ClNO2	452.11	13.52%
T5750	Oxyberberine	549-21-3	C20H17NO5	351.35	13.52%
T6S0657	Isorhyncophylline	6859-01-4	C22H28N2O4	384.47	13.51%
T6S0781	Phellodendrine	6873-13-8	C20H24NO4+	342.41	13.49%
T5800	Allosecurinin	884-68-4	C13H15NO2	217.26	13.47%
T3243	Betaine	107-43-7	C5H11NO2	117.15	13.46%
T3S1888	Deltaline	6836-11-9	C27H41NO8	507.62	13.45%
T3S2340	Usaramine	15503-87-4	C18H25NO6	351.39	13.33%
T3324	Lycorine	476-28-8	C16H17NO4	287.31	13.31%
T6S0659	Rhynchophylline	76-66-4	C22H28N2O4	384.47	13.31%
T5S2102	Leonurine	24697-74-3	C14H21N3O5	311.33	13.29%
T5S0661	Koumine	1358-76-5	C20H22N2O	306.4	13.15%
T3S1320	Magnoflorine iodide	4277-43-4	C20H24INO4	469.31	13.11%
T8206	Cycleanine	518-94-5	C38H42N2O6	622.7	13.08%
T2S2108	Coixol	532-91-2	C8H7NO3	165.14	13.01%
T8120	N-Benzylstearamide	5327-45-7	C25H43NO	373.6	13.01%
T6S1010	Allomatrine	641-39-4	C15H24N2O	248.36	12.99%
T4S0537	Bullatine B	466-26-2	C24H39NO6	437.57	12.98%
T6S0084	Tuberstemonine	6879-01-2	C22H33NO4	375.5	12.98%
T5811	Hyoscine hydrochloride	55-16-3	C17H22ClNO4	339.8	12.98%
PDK0060	Pseudotropine	135-97-7	C8H15NO	141.21	12.98%
T4963	Higenamine	5843-65-2	C16H17NO3	271.31	12.95%
T7557	Guvacine hydrochloride	6027-91-4	C6H10ClNO2	163.6	12.94%
T6569	L-Mimosine	500-44-7	C8H10N2O4	198.18	12.93%
T8161	Vicine	152-93-2	C10H16N4O7	304.26	12.84%
T6S1880	Benzoylaconitine	466-24-0	C32H45NO10	603.7	12.80%
T2142	Yohimbine hydrochloride	65-19-0	C21H27ClN2O3	390.9	12.79%
T2803	Monocrotaline	315-22-0	C16H23NO6	325.36	12.79%
T4953	Neotuberostemonine	143120-46-1	C22H33NO4	375.5	12.79%
T0086	Galanthamine HBr	1953-04-4	C17H21NO3·HBr	368.27	12.78%
T6S1884	Benzoylhypacoitine	63238-66-4	C31H43NO9	573.7	12.75%
T6377	Aloperine	56293-29-9	C15H24N2	232.36	12.70%

T3S0478	Scopolamine	51-34-3	C17H21NO4	303.35	12.70%
T7853	Pachycarpine	492-08-0	C15H26N2	234.38	12.68%
T8016	N-Benzylmethylamine	103-67-3	C8H11N	121.18	12.61%
TN2239	Strictosamide	23141-25-5	C26H30N2O8	498.5	12.55%
T7975	Rhodamine B	81-88-9	C28H31ClN2O3	479.01	12.54%
PDK0014	3-Methylxanthine	1076-22-8	C6H6N4O2	166.1	12.54%
T2S1008	Oxysophoridine	54809-74-4	C15H24N2O2	264.36	12.52%
T8207	Evolitrine	523-66-0	C13H11NO3	229.23	12.48%
T0970	Anisodamine	17659-49-3	C17H23NO4	305.38	12.47%
Fr16741	Oxindole	59-48-3	C8H7NO	133.2	12.44%
T8298	Vasicine hydrochloride	7174-27-8	C11H13ClN2O	224.68	12.41%
T3251	Stachydrine Hydrochloride	4136-37-2	C7H14ClNO2	179.64	12.40%
T3S2105	N-Benzylpalmitamide	74058-71-2	C23H39NO	345.57	12.39%
Fr13711	Tropinone	532-24-1	C8H13NO	139.19	12.39%
T2986	Jatrorrhizine hydrochloride	960383-96-4	C20H20ClNO4	373.83	12.38%
T5543	TriacetonaMine	826-36-8	C9H17NO	155.24	12.38%
T2811	Harmine hydrochloride	343-27-1	C13H13ClN2O	248.71	12.36%
TN1129	Protostemotinine	169534-85-4	C23H29NO6	415.48	12.36%
T2726	Sinomenine	115-53-7	C19H23NO4	329.4	12.33%
T8307	Hydroxy-α-sanshool	83883-10-7	C16H25NO2	263.37	12.32%
T5901	3-Hydroxy-2-methylpyridine	1121-25-1	C6H7NO	109.13	12.31%
T5S0662	Gelsemine	509-15-9	C20H22N2O2	322.4	12.30%
T7044	Norepinephrine	51-41-2	C8H11NO3	169.18	12.28%
TQ0192	Senecionine	130-01-8	C18H25NO5	335.39	12.28%
TN1925	Methyl anthranilate	134-20-3	C8H9NO2	151.1	12.24%
T5S1889	Yunaconitine	70578-24-4	C35H49NO11	659.77	12.23%
T5868	Methyl 3-indolecarboxylate	942-24-5	C10H9NO2	175.2	12.23%
T7846	Aegeline	456-12-2	C18H19NO3	297.3	12.21%
T5693	Methoxatin disodium salt	122628-50-6	C14H4N2Na2O8	374.17	12.21%
T3S1873	Talatisamine	20501-56-8	C24H39NO5	421.57	12.17%
T0052L	Strychnine Sulfate	60-41-3	C21H22N2O2·0·5H2SO4	383.45	12.16%
T5S1882	Napellonine	509-24-0	C22H31NO3	357.49	12.15%
T4S1321	Magnoflorine chloride	6681-18-1	C20H24ClNO4	377.86	12.14%
T10990	Dehydrocorydaline chloride	10605-03-5	C22H24ClNO4	401.88	12.14%
T3158	Harmane	486-84-0	C12H10N2	182.22	12.13%
T5S0106	Peimisine	19773-24-1	C27H41NO3	427.62	12.11%
T0486	Irinotecan hydrochloride trihyd	136572-09-3	C33H45ClN4O9	677.18	12.10%

T0647	Tryptamine	61-54-1	C10H12N2	160.22	12.09%
T6S1885	Benzoylmesaconine	63238-67-5	C31H43NO10	589.7	12.09%
TN1899	Lycoramine	21133-52-8	C17H23NO3	289.4	12.06%
TN3695	Coniine hydrochloride	15991-59-0	C8H18ClN	163.7	12.06%
T5769	Stachydrine	471-87-4	C7H13NO2	143.18	12.05%
T3901	Solasonine	19121-58-5	C45H73NO16	884.07	12.03%
TN6712	Yibeissine	143502-51-6	C27H41NO4	443.6	12.02%
T2S0112	Yibeinoside A	98985-24-1	C33H53NO7	575.8	12.01%
T19792	1,3,7-Trimethyluric acid	5415-44-1	C8H10N4O3	210.19	11.99%
TN1078	Seneciphylline	480-81-9	C18H23NO5	333.39	11.99%
T2890	Hordenine	539-15-1	C10H15NO	165.23	11.98%
T2850	(+)-Bicuculline	485-49-4	C20H17NO6	367.36	11.98%
TL0001	Dencichine	5302-45-4	C5H8N2O5	176.13	11.94%
T20712	Conessine	546-06-5	C24H40N2	356.59	11.94%
T3S1319	Magnoflorine	2141-09-5	C20H24NO4+	342.41	11.89%
T3S0629	Delsoline	509-18-2	C25H41NO7	467.6	11.89%
T2S2335	Dehydroevodiamine	67909-49-3	C19H15N3O	301.34	11.89%
T5S0658	Corynoxine	6877-32-3	C22H28N2O4	384.47	11.89%
T7026	KukoaMine B	164991-67-7	C28H42N4O6	530.66	11.89%
T0130	Physostigmine Salicylate	57-64-7	C22H27N3O5	413.48	11.86%
T4550	Ajmaline	4360-12-7	C20H26N2O2	326.4	11.86%
Fr16605	Lupinine	486-70-4	C10H19NO	169.3	11.86%
T1711	Harmine	442-51-3	C13H12N2O	212.25	11.85%
T3S1227	Aristololactam I	13395-02-3	C17H11NO4	293.27	11.84%
T8287	Sipeimine-3β-D-glucoside	32685-93-1	C33H53NO8	591.8	11.84%
T1591L	Cytidine	65-46-3	C9H13N3O5	243.22	11.83%
T0486L	Irinotecan Hydrochloride	100286-90-6	C33H39ClN4O6	623.14	11.83%
T6981	Nudifloric Acid	3719-45-7	C7H7NO3	153.14	11.83%
T12039	Miglustat	72599-27-0	C10H21NO4	219.28	11.83%
T3S2100	Securinine	5610-40-2	C13H15NO2	217.26	11.80%
T2725	Scopolamine HBr	114-49-8	C17H22BrNO4	384.26	11.79%
T6S0109	Sipeimine	61825-98-7	C27H43NO3	429.64	11.78%
T2797	Harmaline	304-21-2	C13H14N2O	214.27	11.77%
T6S0052	Chelerythrine	34316-15-9	C21H18NO4+	348.37	11.77%
T3S1957	9-Aminocamptothecin	91421-43-1	C20H17N3O4	363.4	11.77%
TN2003	Obtucarbamate A	6935-99-5	C11H14N2O4	238.2	11.77%
T2S0663	Humantenmine	82354-38-9	C19H22N2O3	326.39	11.76%

T3S0128	Hydroprotopine	128397-41-1	C20H20NO5+	354.38	11.75%
T1521	Hydroxy Camptothecine	64439-81-2	C20H16N2O5	364.35	11.71%
T3366	Cephalotaxine	24316-19-6	C18H21NO4	315.37	11.71%
T5805	Norarecoline hydrochloride	6197-39-3	C7H12ClNO2	177.63	11.71%
T0479	Cytisine	485-35-8	C11H14N2O	190.25	11.70%
T4S1619	Hyoscyamine sulfate hydrate	620-61-1	C17H25NO7S	387.44	11.69%
T5143	Corynoxine hydrochloride(687	T5143	C22H29ClN2O4	420.93	11.67%
T8175	Laudanosine	1699-51-0	C21H27NO4	357.4	11.67%
T2921	Sinomenine hydrochloride	6080-33-7	C19H24ClNO4	365.85	11.66%
T6S0654	Isocorynoxeine	51014-29-0	C22H26N2O4	382.46	11.62%
T4S1725	Galanthamine	357-70-0	C17H21NO3	287.35	11.61%
T2182	Scopolamine HBr trihydrate	6533-68-2	C17H28BrNO7	438.31	11.59%
TJS0312	7-hydroxy-4-methyl-8-nitrocou	19037-69-5	C10H7NO5	221.16	11.59%
T3S1729	Dihydrolycorine	6271-21-2	C16H19NO4	289.33	11.59%
T2181	Scopolamine N-oxide HBr	6106-81-6	C17H21NO5·HBr	400.26	11.57%
T3026	(-)-Huperzine A	102518-79-6	C15H18N2O	242.32	11.57%
T5S0994	N-Methylcytisine	486-86-2	C12H16N2O	204.27	11.57%
T5718	L-ABRINE	526-31-8	C12H14N2O2	218.25	11.57%
T0019	Betaine hydrochloride	590-46-5	C5H12ClNO2	153.61	11.56%
T5853	Rubitecan	91421-42-0	C20H15N3O6	393.35	11.56%
T2887	Trigonelline	535-83-1	C7H7NO2	137.14	11.55%
T6S0033	2-Hydroxyadenosine	1818-71-9	C10H13N5O5	283.24	11.51%
T5786	Tetrahydropiperine	23434-88-0	C17H23NO3	289.37	11.48%
T5S0273	Hypaphorine	487-58-1	C14H18N2O2	246.31	11.46%
TWP2911	Thymidine	50-89-5	C10H14N2O5	242.23	11.46%
TWS0704	N-Methylnuciferine	754919-24-9	C20H24NO2+	310.41	11.46%
T2935	Phenethylamine	64-04-0	C8H11N	121.18	11.45%
T4S0536	Bullatine A	1354-84-3	C22H33NO2	343.51	11.44%
T0069	Uracil	66-22-8	C4H4N2O2	112.09	11.43%
T4S1869	12-Epinapelline	110064-71-6	C22H33NO3	359.51	11.43%
T1270	Vincristine sulfate	2068-78-2	C46H58N4O14S	923.04	11.40%
T5595	Tropine	120-29-6	C8H15NO	141.21	11.40%
T6S0630	Hypaconitine	6900-87-4	C33H45NO10	615.72	11.38%
T3A2467	Allocryptopine	485-91-6	C21H23NO5	369.4	11.37%
T6S0627	Mesaconitine	2752-64-9	C33H45NO11	631.71	11.36%
T4080	Leonurine hydrochloride	24735-18-0	C14H22ClN3O5	347.79	11.36%
T5814	Aurantiamide	58115-31-4	C25H26N2O3	402.5	11.36%

T2183	Scopolamine butylbromide	149-64-4	C21H30NO4·Br	440.37	11.33%
T3339	Sophoridine	6882-68-4	C15H24N2O	248.36	11.33%
T3363	Jervine	469-59-0	C27H39NO3	425.61	11.33%
T2947	Indole-3-carbinol	700-06-1	C9H9NO	147.17	11.31%
T6S0107	Peimine	23496-41-5	C27H45NO3	431.66	11.31%
T5S1891	Acetylaconitine	77181-26-1	C36H49NO12	687.77	11.31%
T3S0631	Fuziline	80665-72-1	C24H39NO7	453.6	11.30%
T5S0761	Nitidine chloride	13063-04-2	C21H18ClNO4	383.82	11.29%
T5S1708	Dendrobine	2115-91-5	C16H25NO2	263.38	11.28%
T8196	Arborin	6873-15-0	C16H14N2O	250.29	11.26%
T1062	Capsaicin	404-86-4	C18H27NO3	305.41	11.25%
T6S2356	(+)-Corynoline	18797-79-0	C21H21NO5	367.4	11.25%
T5S0802	Palmatine	3486-67-7	C21H22NO4+	352.4	11.25%
T2S1720	Huperzine B	103548-82-9	C16H20N2O	256.34	11.25%
T0375	Atropine	51-55-8	C17H23NO3	289.37	11.24%
T2S1200	Sinapine	18696-26-9	C16H24NO5+	310.36	11.24%
T4035	Khasianine	32449-98-2	C39H63NO11	721.93	11.24%
T0012	Cinchonine	118-10-5	C19H22N2O	294.39	11.23%
T5S0803	Columbamine	3621-36-1	C20H20NO4+	338.38	11.23%
T5S0055	Chelidonine	476-32-4	C20H19NO5	353.37	11.23%
T6S0105	Peiminine	18059-10-4	C27H43NO3	429.64	11.22%
T1681	Aminophylline	317-34-0	2C7H8O2·C2H8N2	420.43	11.21%
T5799	Arecaidine hydrochloride	6018-28-6	C7H12ClNO2	177.63	11.20%
T0167	Vinpocetine	42971-09-5	C22H26N2O2	350.45	11.18%
T3S1892	Crassicauline A	79592-91-9	C35H49NO10	643.77	11.18%
T0925	Allantoin	97-59-6	C4H6N4O3	158.12	11.17%
T2870	(+)-Matrine	519-02-8	C15H24N2O	248.37	11.16%
T3S0970	Thermopsine	486-90-8	C15H20N2O	244.33	11.16%
T0437	Inosine	58-63-9	C10H12N4O5	268.23	11.14%
T2198	Arecoline HBr	300-08-3	C8H13NO2·HBr	236.11	11.14%
T5061	Lumichrome	1086-80-2	C12H10N4O2	242.23	11.14%
T2993	Cordycepin	73-03-0	C10H13N5O3	251.24	11.13%
T5765	ranaconitine	1360-76-5	C32H44N2O9	600.7	11.10%
T2718	Palmatine chloride	10605-02-4	C21H22ClNO4	387.87	11.08%
T3030	L-Sophoridine	83148-91-8	C15H24N2O	248.36	11.07%
T0804	(+)-Pilocarpine hydrochloride	54-71-7	C11H16N2O2·HCl	244.72	11.06%
T6560	Lappaconite HBr	97792-45-5	C32H44N2O8·HBr	665.61	11.06%

T6S0655	Corynoxeine	630-94-4	C22H26N2O4	382.46	11.05%
T2786	Oxysophocarpine	26904-64-3	C15H22N2O2	262.35	11.01%
T1362	6-Hydroxypurine	68-94-0	C5H4N4O	136.11	11.00%
T7126	Hydroquinine	522-66-7	C20H26N2O2	326.43	11.00%
T3S1967	Abrine	21339-55-9	C12H14N2O2	218.25	10.98%
T2149	Eburnalritardo	4880-88-0	C19H22N2O	294.39	10.95%
T7938	Quinidine	56-54-2	C20H24N2O2	324.42	10.95%
T1083	Theophylline	58-55-9	C7H8N4O2	180.16	10.94%
T2773	Vindoline	2182-14-1	C25H32N2O6	456.54	10.93%
T7054	N-Benzoyl-(2R,3S)-3-phenylis	132201-33-3	C16H15NO4	285.29	10.92%
T0853	Adenosine	58-61-7	C10H13N5O4	267.24	10.91%
T0962	L-Hyoscyamine	101-31-5	C17H23NO3	289.38	10.91%
T2927	Trigonelline hydrochloride	6138-41-6	C7H8ClNO2	173.6	10.90%
T2754	Oxymatrine	16837-52-8	C15H24N2O2	264.37	10.90%
T2825	Cyclopamine	4449-51-8	C27H41NO2	411.63	10.89%
T4952	10-Hydroxycampothecin	67656-30-8	C20H16N2O5	364.35	10.84%
TN2085	Piperlonguminine	5950-12-9	C16H19NO3	273.3	10.84%
T3002	Piperine	94-62-2	C17H19NO3	285.35	10.82%
T0792	(-)-Sparteine sulfate pentahydr	6160-12-9	C15H38N2O9S	422.54	10.82%
T5S1952	9-Methoxycamptothecin	39026-92-1	C21H18N2O5	378.38	10.82%
T3364	Veratramine	60-70-8	C27H39NO2	409.61	10.77%
T4S0111	Hupehenine	98243-57-3	C27H45NO2	415.66	10.74%
T2806	Lappaconitine	32854-75-4	C32H44N2O8	584.64	10.70%
T0934	Nicotinamide	98-92-0	C6H6N2O	122.12	10.69%
T1156	Palonosetron hydrochloride	135729-62-3	C19H24N2O·HCl	332.87	10.61%
T2764	10-Hydroxycamptothecin	19685-09-7	C20H16N2O5	364.36	10.61%
T2217	Cephalomannine	71610-00-9	C45H53NO14	831.91	10.61%
T0186	Docetaxel trihydrate	148408-66-6	C43H59NO17	861.95	10.53%
T2746	Sophocarpine	145572-44-7	C15H22N2O	246.35	10.49%
T5746	Dictamnine	484-29-7	C12H9NO2	199.21	10.49%
T3380	Homoharringtonine	26833-87-4	C29H39NO9	545.61	10.46%
T0190	Vinorelbine	71486-22-1	C45H54N4O8	778.93	10.46%
T1703	SN38	86639-52-3	C22H20N2O5	392.4	10.41%
T4601	9-Methoxycanthin-6-one	74991-91-6	C15H10N2O2	250.3	10.38%
T0320	Colchicine	64-86-8	C22H25NO6	399.44	10.34%
T1174	Topotecan hydrochloride	119413-54-6	C23H24ClN3O5	457.92	10.32%
T1231	Pilocarpine nitrate	148-72-1	C11H16N2O2·HNO3	271.27	10.32%

T1317	Cinchonidine	485-71-2	C19H22N2O	294.39	10.29%
T3S1955	7-Ethylcamptothecin	78287-27-1	C22H20N2O4	376.41	10.28%
T4333	Quinine dihydrochloride	60-93-5	C20H26Cl2N2O2	397.34	10.26%
T0266	Quinidine hydrochloride	6151-40-2	C20H27ClN2O3	378.89	10.20%
T0690	Quinine	130-95-0	C20H24N2O2	324.42	10.20%
T1334	Hydroquinidine	1435-55-8	C20H26N2O2	326.43	10.10%
TN1305	6-Ethoxydihydrosanguinarine	28342-31-6	C22H19NO5	377.39	10.08%
T8724	6-Methoxydihydrosanguinarin	72401-54-8	C21H17NO5	363.36	10.06%
TN6705	Graveoline	485-61-0	C17H13NO3	279.29	10.06%
T7939	Fingolimod	162359-55-9	C19H33NO2	307.47	9.99%
T2774	Lycorine chloride	2188-68-3	C16H18ClNO4	323.17	9.90%
T8892	Emetine	483-18-1	C29H40N2O4	480.6	9.90%
T7056	Dronedarone	141626-36-0	C31H44N2O5S	556.76	9.83%
T6947	Piperlongumine	20069-09-4	C17H19NO5	317.34	9.80%
T4034	Solamargine	20311-51-7	C45H73NO15	868.07	9.79%
T1123	(S)-(+)-Camptothecin	7689-03-4	C20H16N2O4	348.36	9.74%
T8286	Harringtonine	26833-85-2	C28H37NO9	531.59	9.74%
T2972	Rutaecarpine	84-26-4	C18H13N3O	287.32	9.70%
T3786	Tomatine	17406-45-0	C50H83NO21	1034.2	9.56%
T0129	Sanguinarine chloride	5578-73-4	C20H14ClNO4	367.78	9.46%
TN1012	Febrifugine	24159-07-7	C16H19N3O3	301.34	9.44%

Note: HT1080 cells were treated with RSL3 (2 μM) + testing compound (5 μM) for 24 h then cell viability assayed with CCK-8 Kit.
