# Supplementary figures and images for: Identification of a group of bisbenzylisoquinoline (BBIQ) compounds as ferroptosis inhibitors
Source: Cell Death Dis. 2022 Nov 26;13(11):1000. doi: 10.1038/s41419-022-05447-8 (PMC9701226; doi:10.1038/s41419-022-05447-8)

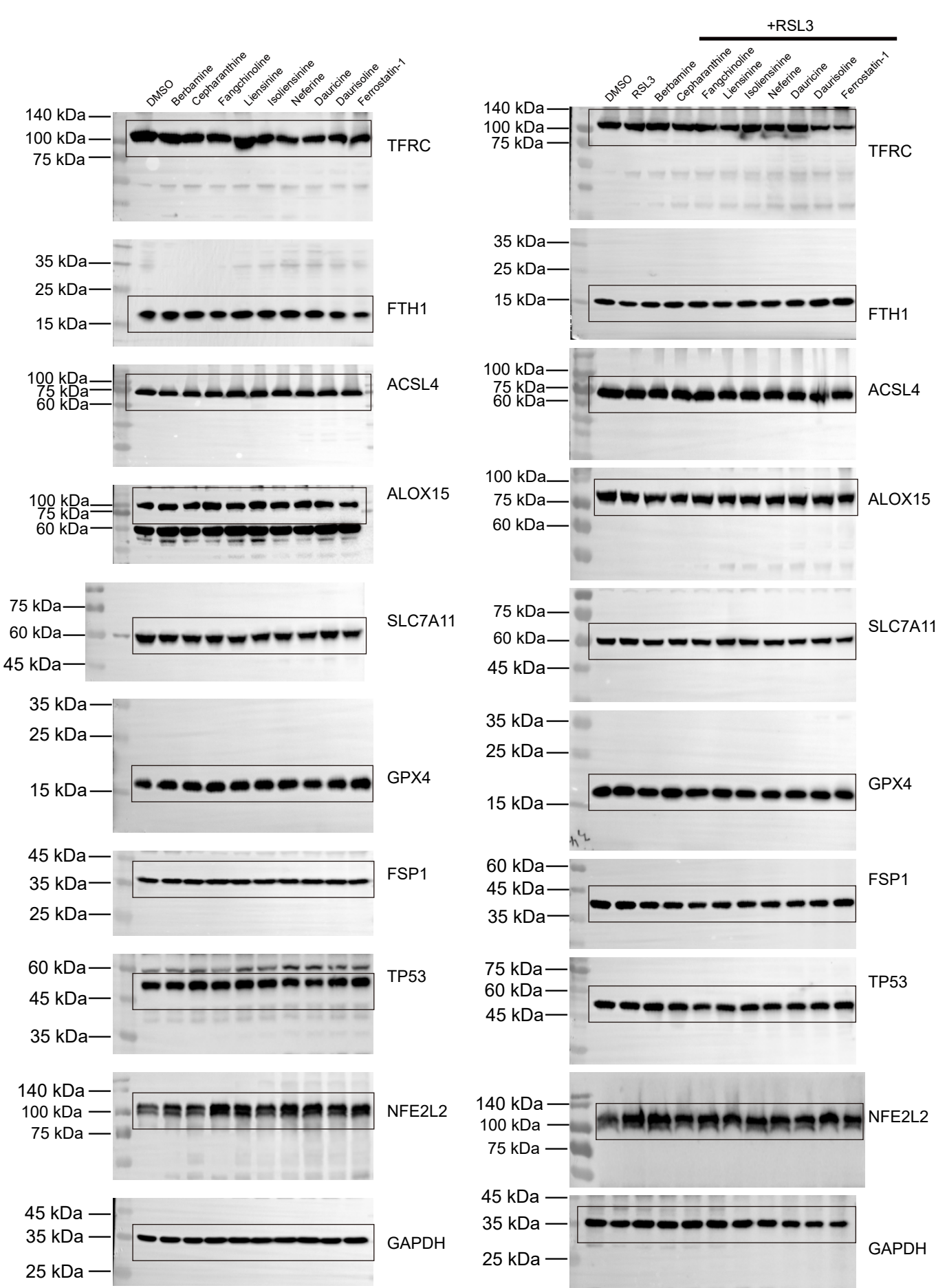

Supplement: Supplementary file 3 — Original western blots [file 41419_2022_5447_MOESM3_ESM.pdf]
